# Photo-responsive polymeric micelles for the light-triggered release of curcumin targeting antimicrobial activity

**DOI:** 10.3389/fmicb.2023.1132781

**Published:** 2023-04-20

**Authors:** Jeffersson Krishan Trigo-Gutierrez, Italo Rodrigo Calori, Geovana de Oliveira Bárbara, Ana Claudia Pavarina, Renato Sonchini Gonçalves, Wilker Caetano, Antonio Claudio Tedesco, Ewerton Garcia de Oliveira Mima

**Affiliations:** ^1^Laboratory of Applied Microbiology, Department of Dental Materials and Prosthodontics, School of Dentistry, São Paulo State University (UNESP), Araraquara, Brazil; ^2^Department of Chemistry, Center of Nanotechnology and Tissue Engineering, Photobiology and Photomedicine Research Group, Faculty of Philosophy, Sciences and Letters of Ribeirão Preto, University of São Paulo, Ribeirão Preto, São Paulo, Brazil; ^3^Department of Chemistry, Research Nucleus of Photodynamic Therapy, State University of Maringá, Maringá, Paraná, Brazil

**Keywords:** Photochemotherapy, micelles, light, bacteria, fungi

## Abstract

Nanocarriers have been successfully used to solubilize, deliver, and increase the bioavailability of curcumin (CUR), but slow CUR release rates hinder its use as a topical photosensitizer in antimicrobial photodynamic therapy. A photo-responsive polymer (PRP) was designed for the light-triggered release of CUR with an effective light activation-dependent antimicrobial response. The characterization of the PRP was compared with non-responsive micelles comprising Pluronics™ P123 and F127. According to the findings, the PRP formed photo-responsive micelles in the nanometric scale (< 100 nm) with a lower critical micelle concentration (3.74 × 10^−4^ M^−1^, 5.8 × 10^−4^ M^−1^, and 7.2 × 10^−6^ M^−1^ for PRP, F127, P123, respectively, at 25°C) and higher entrapment efficiency of CUR (88.7, 77.2, and 72.3% for PRP, F127, and P123 micelles, respectively) than the pluronics evaluated. The PRP provided enhanced protection of CUR compared to P123 micelles, as demonstrated in fluorescence quenching studies. The light-triggered release of CUR from PRP occurred with UV light irradiation (at 355 nm and 25 mW cm^−2^) and a cumulative release of 88.34% of CUR within 1 h compared to 80% from pluronics after 36 h. *In vitro* studies showed that CUR-loaded PRP was non-toxic to mammal cell, showed inactivation of the pathogenic microorganisms *Candida albicans*, *Pseudomonas aeruginosa*, and methicillin-resistant *Staphylococcus aureus*, and decreased biofilm biomass when associated with blue light (455  nm, 33.84 J/cm^2^). The findings show that the CUR-loaded PRP micelle is a viable option for antimicrobial activity.

## 1. Introduction

Antimicrobial resistance is a current global severe health problem. Along with biofilm-associated illnesses, microbial resistance may cause the premature death of 300 million people over the next 35 years if it is not overcome (Morrison and Zembower, 2020). Antimicrobial resistance refers to the overuse and misuse of conventional antimicrobial drugs (antibiotics or antifungals) - the first option in treating human and veterinarian infectious disease (McEwen and McEwen and Collignon, 2018). Moreover, biofilm is a complex community of microbial cells attached to a surface and embedded in a self-produced extra-polymeric matrix that forms a physical barrier to protect microbial cells from external agents and the host defense. As biofilms, microorganisms show different features from free-floating (planktonic) cells, such as increased tolerance to antimicrobial agents (Ciofu et al., 2022).

One of the most popular multidrug-resistant microorganisms is the Gram-positive bacterium methicillin-resistant *Staphylococcus aureus* (MRSA), which is associated with skin infections, pneumonia, osteomyelitis, sepsis, and bacteremia (Lee et al., 2018). Moreover, such microorganism has been associated with community-acquired and hospital-acquired (nosocomial) infections (Jubeh et al., 2020). Apart from MRSA, the World Health Organization has also listed *Pseudomonas aeruginosa* (Gram-negative bacterium) as “critical,” because it can be deadly for immunosuppressed patients (World Health Organization, 2017). Additionally, this bacterium is associated with lung diseases (Bisht et al., 2020) and nosocomial infection (Kollef et al., 2021).

Besides bacteria, fungi from the *Candida* genus are associated with bloodstream infections (Martinez and Wolk, 2016; Raja, 2021). The opportunistic fungus *Candida albicans* (a commensal species of the human body) is responsible for local and systemic infections, such as oral and vaginal candidiasis and candidemia. *C. albicans* grows as yeast and filamentous forms (pseudohyphae and hyphae) (Nobile and Johnson, 2015; Pappas et al., 2018), the last one is the pathogenic form and responsible for local infections due to the ability to invade the epithelium (Chen et al., 2020). The infections caused by these pathogenic microorganisms require novel and efficient treatment strategies (Koo et al., 2017).

Antimicrobial photodynamic therapy (aPDT) has been suggested as an alternative and/or adjunctive to conventional antimicrobial drugs in the fight against microbial resistance and biofilms (Muehler et al., 2020). The aPDT combines a photosensitizer (PS) compound, molecular oxygen, and light to inactivate microorganisms (Hamblin and Abrahamse, 2020; Muehler et al., 2020; Warrier et al., 2021). In practical terms, the PS is applied to microorganisms for some time (pre-irradiation time, PIT) for its uptake. Then, the target microorganisms are irradiated with a light of a suitable wavelength (the same absorption wavelength of the PS). The PS is excited producing reactive oxygen species (ROS); or energy transfer to molecular oxygen, forming singlet oxygen (^1^O_2_), which is the main reactive species of aPDT. The formation of those cytotoxic species *in situ* promotes microbial death by oxidative damage (Hamblin and Abrahamse, 2020; Muehler et al., 2020; Warrier et al., 2021).

The natural compound curcumin (CUR), isolated from the rhizome of *Curcuma longa* L. (Pulido-Moran et al., 2016), has been used as a promising PS in aPDT. Despite the history of success against fungi, bacteria, and viruses (Dovigo et al., 2011a,b; Quishida et al., 2016; Trigo-Gutierrez et al., 2021), its instability and low bioavailability (Priyadarsini, 2014) have imposed drawbacks to its clinical application. Moreover, the hydrophobic nature of the PS can cause intracellular aggregations that quench its photoactivity in PDT (Calori and Tedesco, 2020). Although organic solvents have been used to provide suitable CUR solubilization, their cytotoxic effect hinders their use (Hanslick et al., 2009; Trigo Gutierrez et al., 2017). As a viable alternative, nanocarriers such as polymeric nanoparticles, emulsions, liposomes, mesoporous nanoparticles, supramolecular complexes, and micelles have been proposed to improve CUR solubility and biocompatibility (Trigo-Gutierrez et al., 2021). In this context, polymeric micelles have been used due to their biocompatibility, physicochemical properties, and easy of preparation (Qiu et al., 2021).

In our previous studies, CUR-loaded polymeric nanoparticles showed reduced microbial photo-inactivation compared with free CUR (Trigo Gutierrez et al., 2017; Sakima et al., 2018). Such reduced efficacy may be due to the slow release of CUR from polymeric nanoparticles (Trigo Gutierrez et al., 2017; Sakima et al., 2018), which prolongs the PIT and may prevent its clinical use as a topical agent. Therefore, stimuli-responsive nanocarriers have been developed to increase the drug-delivery at the target site, which is interesting for topical applications, such as biofilm-related infections. Temperature, pH, level of ROS, ultrasound, magnetic field, and light are used as exogenous or endogenous stimuli, and the majority of stimuli-responsive micelles used for CUR are pH-sensitive (Qiu et al., 2021). A photo-responsive nanocarrier as a light-triggered release system would be suitable for CUR-mediated aPDT, allowing a spatial–temporal release during light irradiation and enhancing therapy efficacy (Fomina et al., 2012). Therefore, a light-responsive nanosystems for CUR would be promising for aPDT against biofilms and resistant microorganisms responsible for infections.

**Figure 1:**
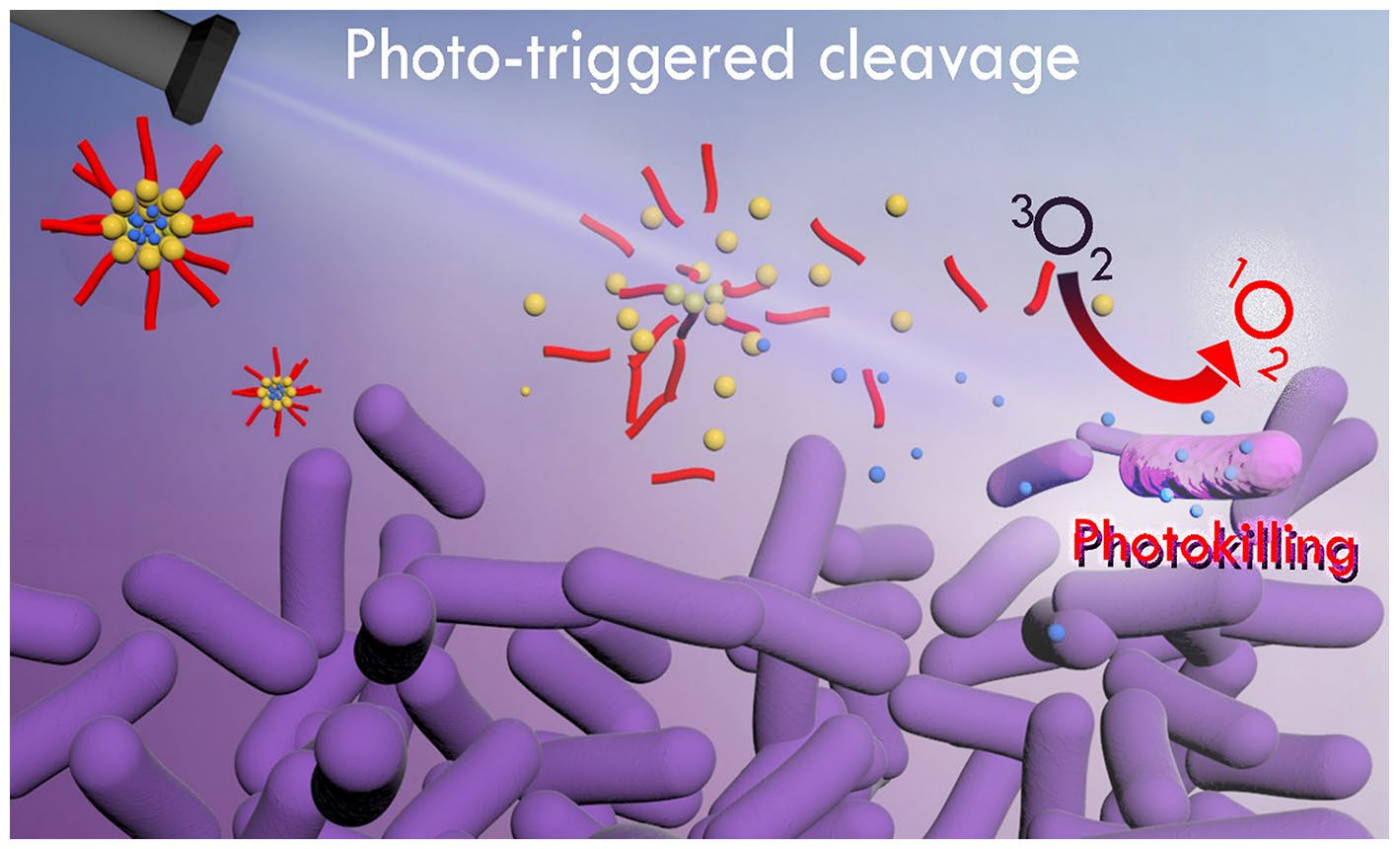
Schematic representation of photo-release of CUR from photo-responsive micelles for photoinactivation of bacterial cells.

Regarding aPDT studies with CUR loaded in photo-responsive nanoparticles, upconverting nanoparticles (UCNP) conjugated with CUR have shown promising results in *in vitro* assays and animal models of infection (Ye et al., 2014; Liu et al., 2018). UCNP are composed of lanthanide and actinide (rare-earth metals) dopants, which converts near infrared (650 to 900 nm, NIR) light into UV–visible light (Yuan et al., 2022). Despite the current trend of using NIR because of its deeper penetration (up to 10 cm) into the host tissue without injury, UCNP shows photothermal effect (Kandoth et al., 2021; Yuan et al., 2022), which may harm the host tissue at the site of infection (Kandoth et al., 2021). Another mechanism of light-responsive drug delivery is the photochemical activation, which is triggered by UV or visible light (Yuan et al., 2022). In spite of the evolution of aPDT and the recent description of innovative technologies for antimicrobial strategies (Youf et al., 2021), to the best of our knowledge, the description of a light-responsive micelle for CUR to be used in aPDT against pathogenic species and biofilms (Figure 1) is not known.

In this study we aimed to synthesize a photo-responsive polymer (PRP) by conjugating poly(ethylene glycol) with the 4-bromomethyl-3-nitrobenzoic group, followed by grafting phenoxyacetic acid, which can self-assemble into photo-responsive micelles in an aqueous medium for the light-triggered release of CUR (Figure 1). After the synthesis, its properties and efficacy in aPDT were compared to free CUR and conventional micelles formed by two of the most used triblock pluronics (F127 and P123) comprised of poly(ethylene oxide) and poly(propylene oxide) blocks arranged as (EO)x-(PO)y-(EO)x. F127 and P123 provided suitable comparative parameters, because they provide micelles with highly different degrees of hydrophobicity due to their structural differences. They present a similar number of central hydrophobic PPO units (65 and 69 for F127 and P123, respectively) (Alexandridis et al., 1994), but differ primarily in terminal hydrophilic PEO moieties (100 and 19 for F127 and P123, respectively). Therefore, P123 micelles are much more hydrophobic than F127, which has been explored to efficiently load PS of quite different hydrophobicity (Alexandridis et al., 1994). When analyzing such pluronics, the loading capacity and biological efficacy of the photo-responsive synthetic polymer could be well evaluated comparatively.

## 2. Materials and methods

### 2.1. Materials

CUR (MW 368.38 g/mol, ≥65% purity); Pluronic P123 (MW 5750 g/mol), Pluronic F127 (MW 12600 g/mol); poly(ethylene glycol) (PEG, MW 1500); 4-bromomethyl-3-nitrobenzoic acid (BNA); 1-ethyl-3-(3-dimethylaminopropyl)carbodiimide (EDC); 4-dimethylaminopyridine (DMAP); dichloromethane (DCM), sodium bicarbonate (NaHCO3); diethyl ether; phenoxyacetic acid (PAA); N, N-dimethylformamide (DMF); N, N-diisopropylethylamine (DIPEA); pyrene (MW 202.25 g/mol, 98% purity); dialysis membrane (cut-off 14 kDa); penicillin; streptomycin; 2,3-bis(2-methoxy-4-nitro-5-sulfophenyl)-2H-tetrazolium-5-carboxanilide (XTT salt); and 1-(4, 5-dimethylthiazol-2-yl)-3, 5-diphenyl-formazan (MTT) were purchased from Sigma-Aldrich (St.Louis, MO, USA).

Anhydrous ethanol of analytical grade was acquired from J.T. Becker™. Anhydrous calcium sulfate, potassium iodide (KI), and isopropanol were acquired from Labsynth (Diadema, SP, Brazil). Sabouraud dextrose agar (SDA, Acumedia Manufacturer Inc., Baltimore, MD), Mannitol Salt Agar (MNA, Difco, Detroit, Michigan, USA), Tryptic Soy Agar (TSA, Difco, Detroit, Michigan, USA), Roswell Park Memorial Institute (RPMI 1640) medium, and Tryptic Soy Broth (TSB) were used as culture media in the microbiological assays. Dulbecco’s modified Eagle’s medium (DMEM), fetal bovine serum (FBS), and glutamine were acquired from GIBCO/BRL (Grand Island, NJ, USA).

The desiccator used in PRP synthesis and the microsyringe used in CMC tests were manufactured by Uniglass (Orem, UT, USA) and Thermo Fischer (Waltham, MA, USA), respectively. Ultrapure water was obtained through the Direct-Q™ 3 water system (Millipore™) and used in all experiments.

Absorption spectra data were collected with a UV–visible spectrophotometer (Ultraspec 7000, GE Lifesciences, Marlborough, MA, USA) from 200 nm to 700 nm. Fluorescence spectra were determined with a spectrofluorometer (Fluorog-3, Horiba Instruments, Edison, NJ, USA).

Dynamic light scattering and zeta potential were performed in a Zetasizer Nano ZS apparatus (Malvern PCS Instruments, UK). The hydrogen nuclear magnetic resonance (^1^H NMR) was performed on an NMR spectrophotometer (Bruker Avance III HD, Billerica, MA, USA). Images were acquired with a field emission scanning electron microscope (FE-SEM, JEOL, JSM-7500F Peabody, MA, USA) and the PC-SEM v 2.1.0.3 operation software. A sputter coater apparatus (BAL-TEC SCD 050, Bridgend, UK) was used to coat the samples with gold. The photo leakage of the PRP was performed with laser light (Continuum, Quantum Composers, Bozeman, MT, USA) at 355 nm and 25 mW/cm^2^. Two light sources were used for the antimicrobial assays: a UV light apparatus (Prizmatix, San Francisco, CA, USA) at 369 nm and 25 mW/cm^2^ for the photo-release of CUR from PRP and a red-green-blue LED light named Biotable (RGB, MMOptics, São Carlos, SP, Brazil) for aPDT (450 nm, 47 m/Wcm^2^). A microplate reader (EZ Read 400, Biochrom, Cambridge, UK) was used for reading absorbance data in the cytotoxic assay.

### 2.2. Synthesis of photo-responsive polymer

#### 2.2.1. Synthesis of 4-bromomethyl-3-nitrobenzoic acid-conjugated PEG_1500_

**Figure 2:**
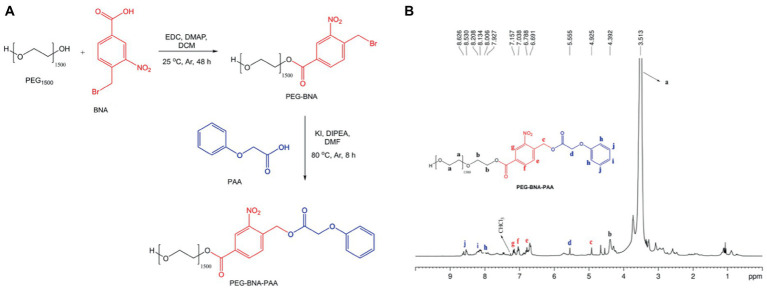
**(A)** Synthetic PRP route. **(B)** NMR spectra from the photosensitive polymer (PEG-BNA-PAA).

The PEG_1500_ polymer with BNA was activated *via* Steglich esterification (Neises and Steglich, 1978) in 1-EDC and DMAP (Figure 2A). A BNA solution (0.35 g, 1.33 mmol) in DCM (20.00 mL) remained under light protection and an argon (Ar) atmosphere. Immediately, EDC (0.33 g, 2.1 mmol) and DMAP (0.12 g, 2.0 mmol) were added and the solution remained under magnetic stirring at room temperature. Next a PEG_1500_ stock solution (1.0 g, 0.67 mmol) diluted in DCM (30 mL) was added by a dripping technique for 45 min. The resulting solution remained under magnetic stirring for 48 h at room temperature and in an Ar atmosphere. Then, the solution was washed three times with 10% NaHCO_3_, and the organic phase was collected and dried with anhydrous calcium sulfate. Immediately, the solution was filtered and the solvent was eliminated by reduced pressure to obtain a yellow pasty product, which was purified by recrystallization with diethyl ether and left in a desiccator for 24 h to eliminate solvent residues. The final product was a solid yellow crystal.

#### 2.2.2. Synthesis of phenoxyacetic acid-conjugated PEG-BNA

The PAA molecule at the end of the PEG-BNA backbone was conjugated by bimolecular nucleophilic substitution (SN2) to obtain ether as a final product (Figure 2A). In a two-necked reaction flask connected to a backflow system with PEG-BNA solution (0.5 g, 3.44 mmol) diluted in DMF (15.0 mL), a solution containing PAA (0.52 g, 3.44 mmol), DIPEA (60 μL, 3.44 mmol), and KI (57.0 mg, 0.34 mmol) was added. The final solution remained under magnetic stirring at 80°C and Ar atmosphere. The reaction was monitored with thin layer chromatography, in which a silica gel plate was used as a stationary phase and a mixture of DCM:methanol (80:20, v/v) as a mobile phase. After 8 h of reaction, the solvent was eliminated by reduced pressure, and the final product was solubilized in DCM and washed three times with 10% NaHCO_3_. The organic phase was collected and dried with anhydrous calcium sulfate. The final product was purified by recrystallization with diethyl ether and left in a desiccator for 24 h to eliminate solvent residues. The final product was a solid reddish crystal. Figure 2A illustrates the synthesis reaction.

#### 2.2.3. Determination of critical micellar concentration

A stock solution of 1% w/v of PRP in water was dropped *via* microsyringe on a 10-mm path-length quartz cuvette containing 2 mL of pyrene aqueous solution (1.0 × 10^−6^ mol/L) as a probe dye. After each increment, the fluorescence spectra were collected at excitation/emission of 330/378 nm (Ding et al., 2016). The CMC of the PRP was also determined with CUR, at excitation/emission of 460/520 nm. For both dyes, CMC values were determined at 22°C, 25°C, 30°C, and 35°C. The same method was used for Pluronics P123 and F127 (1 and 4% w/v, respectively) at 25°C and 37°C with pyrene or CUR.

### 2.3. Synthesis and characterization of micelles

The PRP and conventional micelles were synthesized by the direct addition method. For PRP micelles synthesis, 114 μL of PRP in a 1% aqueous solution was added to 1.886 μL of an aqueous:acetonitrile solution with CUR at 5.30 × 10^−6^ mol/L. For F127 micelles, CUR at 5.33 × 10^−6^ mol/L solubilized in 1.870 μL of aqueous:acetonitrile was added to 130 μL of the pluronic stock solution at 4%. Finally, for the Pluronic P123 micelles, CUR at 6.75 × 10^−6^ mol/L in an aqueous:acetonitrile solution was added to 520 μL of P123 in a 1% aqueous solution. The CMC of PRP and Equilibrium Constant (Ks) of conventional micelles determined these values. CUR-free micelles were also synthesized with the same procedure used for PRP and conventional micelles. After the synthesis, the hydrodynamic size, polydispersity index (PdI), and zeta potential were determined by DLS at 25°C and a scattering angle of 173°. A volume of 2 mL of undiluted samples of each CUR-loaded micellar suspension or CUR-free micelles was dropped in a DLS cuvette.

#### 2.3.1. Entrapment efficiency (EE%)

After micelle synthesis, the absorbance of each synthesized micelle was measured spectrophotometrically (A0). Immediately after, the sample was centrifuged at 26,600 *xg* and 25°C for 10 min (Sahu et al., 2011). The pellet was resuspended in acetonitrile and absorbance was measured again (A1). The difference between A0 and A1 was used in the regression equation (*R*^2^ > 0.99) obtained from a calibration curve to determine CUR concentration. The EE% was determined by the following formula (Sahu et al., 2011):

(1) $$EE\%=\frac{Encapsulatedamount}{Totalamountof\;CUR}*100$$

#### 2.3.2. Equilibrium constant

The equilibrium constant (Ks) was determined with successive additions of an aqueous stock solution of PRP at 1% w/v into a quartz cuvette with 2 mL of a water solution of CUR (5.00 × 10^−6^ mol/L). After each increment, the absorption spectra of the solution were measured at 425 nm. Additional samples of an aqueous solution of Pluronic F127 at 4% w/v or P123 at 1% w/v were also used to determine the Ks of CUR in each pluronic. In all cases, the tests were performed at 25°C. The binding constant (K_b_) can be described by the equilibrium, as follows (Boruah et al., 2012):

(2) CUR+polymer↔[CUR−polymer]

The following equation (Boruah et al., 2012) determined the equilibrium constant (Ks):

(3) $$Ks=\frac{\left[complex\;CUR↔polymer\right]}{\left[CUR\right]\left[polymer\right]}$$

where, [complex CUR↔polymer] is the interaction between CUR and the polymer, [CUR] is the CUR concentration, and [polymer] is the polymer concentration. The linear plot was used assuming the formation of 1:1 between CUR and the polymer, then the Ks was determined as follows (Boruah et al., 2012):

(4) $$\frac{1}{\Delta A}=1/Ks\Delta \varepsilon \left[CUR\right]\left(\frac{1}{\left[polymer\right)}+\frac{1}{\Delta \varepsilon \left[CUR\right]}\right]$$

where, ∆*A* is the absorbance change, ∆*ε* is the molar extinction coefficient at a wavelength of 425 nm, and [CUR] and [polymer] are the equilibrium concentrations of curcumin and polymer, respectively.

#### 2.3.3. Fluorescence quenching

A stock solution of 2 mM sodium iodide (NaI) was titrated in each CUR-loaded micellar suspension (PRP and Pluronics F127 and P123). Immediately after, fluorescence was measured. The Stern-Volmer plot was performed to obtain the quenching constants, as follows (Calori and Tedesco, 2016):

(5) $$\frac{F0}{F}=1+Ksv\;\left[Q\right]$$

where *F* 0 and *F* are the fluorescence intensities of CUR in the absence and presence of a quencher, respectively; KSV is the Stern-Volmer constant; [Q] is the quencher concentration. For Stern-Volmer deviation, the modified equation was used, as follows (Kumar et al., 2016):

(6) $$\frac{F0}{\Delta F}=\frac{1}{faK^{\prime }D\left[Q\right]}+\frac{1}{fa}$$

where ∆*F* = *F* 0 - F is the difference between initial fluorescence intensity and fluorescence of CUR-loaded micelles in the quencher [*Q*] at any point in the quenching titration, *f*_a_ is the fraction of total CUR relative to the quencher, and K’_*D*_ is the Stern–Volmer constant.

#### 2.3.4. Polymer photo-response

Samples of PRP with pyrene at 1.0 × 10^−6^ mol/L or CUR at 5.0 × 10^−6^ mol/L were subjected to a UV light by pulsed laser irradiation at 25 mW/cm and 355 nm. The samples were irradiated at fluences of 9, 18, 27, 54, 72, and 90 J/cm (Ding et al., 2016). After each irradiation, the absorbance of PRP micelles was measured spectrophotometrically from 200 nm to 600 nm.

#### 2.3.5. CUR photo-release

Samples of PRP at 0.057% w/v containing CUR at 5.0 × 10^−6^ mol/L were irradiated with the same parameters as the photo-response assay. After each irradiation, the samples were centrifuged (26,600 *xg* for 10 min at 25°C). The CUR released was quantified with a standard curve of CUR ranging from 7.0 × 10^−7^ mol/L to 4.9 × 10^−6^ mol/L serially double diluted (*R*^2^ ≥ 0.99).

#### 2.3.6. CUR release from conventional micelles

Samples of CUR-loaded micelles (50 mL) were individually added to a dialysis membrane and submerged in a phosphate buffer solution (PBS). The samples were treated under magnetic stirring at 100 rpm at 37°C. An aliquot of dialyzed solution (PBS and free CUR) was removed at time intervals (0, 15, and 30 min and 1, 2, 4, 6, 8, 10, 12, 18, 24, and 36 h) and centrifuged at 26,600 *xg* and 25°C for 10 min. The supernatant was solubilized in acetonitrile and its absorbance was measured at 425 nm. The CUR concentration was determined with a standard curve (7.00 × 10^−7^ mol/L to 4.90 × 10^−6^ mol/L serially double diluted, *R*^2^ > 0.99). The release of CUR from micelles (PRP or conventional) was determined with the following equation (Sahu et al., 2011):

(7) $$CUR\;release\;\left(\%\right)=\frac{Amountof\;CUR\;released}{Totalamountof\;CUR}*100\;$$

### 2.4. Cytotoxicity assay

Spontaneously immortalized normal oral keratinocytes (NOK-si, kindly provided by Professor Carlos Rossa Junior, Department of Diagnosis and Surgery, School of Dentistry, Araraquara, UNESP, Brazil) were grown in DMEM with 10% fetal bovine serum, 2 mM glutamine, and 100 UI/mL and 100 mg/mL of penicillin and streptomycin, respectively, at 37°C under 5% CO_2_, 80% humidity, up to 90% confluence. A total of 20,000 cells were seeded in a 96 flat-bottom plate and incubated for 24 h to form a monolayer cell surface (Trigo Gutierrez et al., 2017). Next, the wells were washed once with PBS, and CUR-loaded or CUR-free PRP or conventional micelles were added immediately. Then, the samples were incubated for 96 min for PRP and 32 min for conventional micelles. Additional samples were incubated with free CUR diluted in ACN or ACN in DMEM to evaluate the effect of the organic solvent alone in mammalian cell culture. The effect of UV light irradiation (369 nm for 1 h, 90 J/cm^2^) on cells was also evaluated. Triton X-100 was used as positive control and culture medium (DMEM) was used as negative control. In all cases, the PS was incubated for the time equivalent to PIT and irradiation time used in aPDT. After incubation, the suspension was aspirated from the wells, the cells were washed once with PBS, and an MTT solution was poured immediately into the wells. The plates were incubated at 37°C for 4 h. Next, the MTT solution was aspirated from the wells, and isopropanol was added to the wells to solubilize MTT crystals. Then, MTT absorbance was measured at 570 nm with a microplate reader (Kumar et al., 2018).

### 2.5. Antimicrobial assays

#### 2.5.1. Microbial strains, growth conditions, and Photoinactivation

Standard strains of *C. albicans* (SC5314), MRSA (ATCC 33591), and *P. aeruginosa* (ATCC 27853) were individually seeded in a specific agar plate for each microorganism. The SDA with 0.05 g/L of chloramphenicol was used for *C. albicans*, MSA was used for MRSA, and TSA was used for *P. aeruginosa*. The plates were incubated for 48 h at 37°C. After incubation, 5–10 colonies of each microorganism were individually transferred to specific broth media: RPMI 1640 (with glutamine and red phenol, without bicarbonate) buffered with MOPS, a pH of 7.0, supplemented with 2% D-glucose for *C. albicans*, and TSB for both bacteria. After overnight incubation at 37°C, each microbial suspension was diluted in fresh broth (1:10 and 1:20) and absorbance was read at 450 nm (for yeast) and 600 nm (for bacteria). Microbial suspensions were standardized by incubating them at 37°C until absorbance reached the mid-log growth phase determined previously for each species. The microbial suspensions were standardized at 4 × 10^6^ (±7 × 10^5^) colony-forming units per milliliter (CFU/mL) for fungi, 3 × 10^7^ (±2 × 10^6^) CFU/mL for MRSA, and 5 × 10^7^ (±4 × 10^6^) CFU/mL for *P. aeruginosa*.

For aPDT against planktonic cultures, 100 μL of each standardized microbial suspension was transferred to wells of a 96-well flat-bottom microtiter plate. The same volume of CUR-loaded PRP, F127, P123 micelles or free CUR was added. Additional wells with microbial suspensions were incubated with blank micelles or PBS. The samples remained in the dark for 20 min (PIT). The PRP micelles were irradiated with a UV light (369 nm) for 1 h (90 J/cm^2^) to release CUR and then illuminated with blue light (455 nm) for 12 min (33.84 J/cm^2^) for PS excitation. The samples incubated with conventional micelles and free CUR did not receive the UV light and were irradiated only with blue light. Supplementary Chart S1 summarizes the experimental condition and light fluence used in each group. Next, each sample was submitted to tenfold serial dilution in PBS, and aliquots of 25 μL were spread on specific agar media for each microorganism. The agar plates were incubated at 37°C for 48 h for colony quantification.

#### 2.5.2. ROS production

The production of ROS after aPDT against planktonic cultures was measured using 2′,7′-dichlorofluorescein diacetate (DCFHDA, Sigma-Aldrich). Initially, DCFHDA at 5 mM (Parasuraman et al., 2019) was incubated with each microbial sample for 30 min. However, the fluorescent intensity of non-treated microbial cells (controls) was higher than those submitted to aPDT (Supplementary Table S1). Similar results were obtained after centrifuging the microbial cells incubated with DCFHDA (Supplementary Table S1). Hence, we performed the experiment reducing the DCFHDA concentration. Each microbial species grown as planktonic cultures was incubated with DCFHDA at 10 μM for 1 h (Mushtaq et al., 2022). The samples were then submitted to aPDT with each photosensitizer (free CUR and CUR loaded in F127, P123, and PRP micelles) as previously described. After illumination, samples were transferred to a black well plate and the fluorescence intensity was measured at excitation/emission of 485 nm/520 nm using a microplate reader (FLUOstar Omega, BMG Labtech, Germany). Planktonic samples incubated with DCFHDA and treated only with the photosensitizers or water in the dark were used as controls. Experiments were performed five times and fluorescence intensity of cells without DCFHDA was used as blank.

#### 2.5.3. Biofilm formation and photoinactivation

A volume of 200 μL of the standardized microbial suspension was individually transferred to wells of a 96-well flat-bottom plate and incubated at 37°C for 90 min (adhesion phase). For removing the non-adherent cells, each well was carefully washed twice with 200 μL of PBS. Next, 200 μL of free CUR or CUR-loaded micelles diluted in specific culture media was transferred to each well. Additional microbial samples were treated with culture media with unloaded micelles and culture media without PS (controls). The plates were incubated at 37°C for 24 h. Then, the culture media were removed from each well and replaced with fresh media containing its respective PS (free CUR or CUR-loaded or unloaded micelles). The plates were incubated again at 37°C for 24 h to complete biofilm formation.

After biofilm growth, the samples were washed twice with PBS and treated with the same groups evaluated in planktonic cultures. The effects of aPDT on the metabolic activity of biofilms and biofilm biomass were assessed with XTT and crystal violet staining, respectively.

For the XTT assay, the suspension of each well was removed after aPDT, and the biofilms were washed twice with PBS. Then, the biofilms were incubated with 200 μL of a solution containing 40 μL of XTT salt at 1 mg/mL diluted in PBS (maintained under −70°C until use), 0.4 mM menadione (2 μL) diluted in acetone (P.A.) and prepared a few minutes before use, and 158 μL of PBS supplemented with 200 mM glucose. Next, the 96-well flat-bottom plate was incubated at 37°C for 3 h in the dark. Then, the absorbance of samples was read spectrophotometrically at 492 nm (Cerca et al., 2005; Trigo-Gutierrez et al., 2018).

In the crystal violet assay, the biofilms were washed twice with PBS, and 200 μL of methanol (80%) was added to each well and maintained for 15 min for biofilm fixation. Next, methanol was removed, and the 1% crystal violet dye was added. After 5 min, the crystal violet was removed, and the biofilm was washed five times with PBS. Subsequently, 33% acetic acid was added to remove the dye from biofilms. Finally, 200 μL of the resulting solution was transferred to a new well of a microtiter plate, and absorbance was read at 570 nm (Trigo-Gutierrez et al., 2018).

#### 2.5.4. PS uptake

Single-species biofilms of MRSA, *P. aeruginosa*, and *C. albicans* were grown onto cover glass-bottom dishes (SPL Life Sciences, Pocheon, Korea) for 48 h with the PS (free CUR and CUR-loaded PRP, F127, and P123 micelles) as described above or without PS (only culture media, untreated control), but using aliquots of 1 mL instead of 200 μL. After incubation, biofilms were washed twice with PBS before being observed under a confocal scanning laser microscope (CSLM, Carl Zeiss LSM 800 with Airyscan, Germany) with laser at 488 nm and objectives of 40x (for bacteria) or 20x (for yeast). The same microscope parameters (laser power at 8%, pinhole of 80 μm, detector gain of 693 V, and detector digital gain of 1.5) were maintained for all samples. The biofilms’ thickness and the fluorescence intensity were recorded for each sample.

### 2.6. Statistical analyses

The data from micelle characterizations were expressed as mean and standard deviation. The data from antimicrobial and cytotoxicity assays were analyzed with ANOVA/Welch and post-hoc Games-Howell tests at a significance level of 5%.

## 3. Results

### 3.1. Synthesis and characterization

The PRP (PEG-BNA-PAA) was synthesized in a two-step synthesis (Figure 2A). First, 4-BNA was conjugated to PEG_1500_ by an amide coupling reaction. Then, PEG-BNA was linked to the PAA forming the PRP. The ^1^H NMR spectroscopy showed proton signals assigned as follows: PEG_1500_ at 3.513 ppm; ester bonds between PEG and BNA at 4.392 ppm; and the bond of the PEG-BNA complex with PAA at 4.925 ppm (Figure 2B), indicating PRP formation.

The direct addition of synthesized PRP (5.0 × 10^−3^ mol/L) in water at 25°C formed micelles. The DLS determined the hydrodynamic diameter (H_D_) as 32.0 (±0.4) nm, showing the nanoscale of the particle. The polydispersity index (PdI) of 0.23 (±0.5) (Supplementary Table S2) showed the formation of micelles in low dispersity. The zeta potential was slightly negative [−0.15 (±0.1) mV].

PRP micelles was also formed in the presence of 5.00 × 10^−6^ mol/L CUR. The H_D_ and PdI increased to 40.4 (±0.8) nm and 0.271 (±0.02). The zeta potential of CUR-loaded PRP micelles was similar to that of CUR-free PRP micelles.

As comparative nanosystems, F127 or P123 micelles in the absence and presence of CUR were prepared, followed by their DLS, and zeta potential evaluations. The DLS showed lower H_D_ for pluronics than PRP micelles (Supplementary Table S2).

#### 3.1.1. Entrapment efficiency (EE%)

The EE% value of CUR in PRP micelles was 89 (±1)% (Supplementary Table S3). The EE% of F127 and P123 was lower than PRP, 78 (±2)% and 73 (±1)%, respectively.

#### 3.1.2. Critical micellar concentration

**Figure 3:**
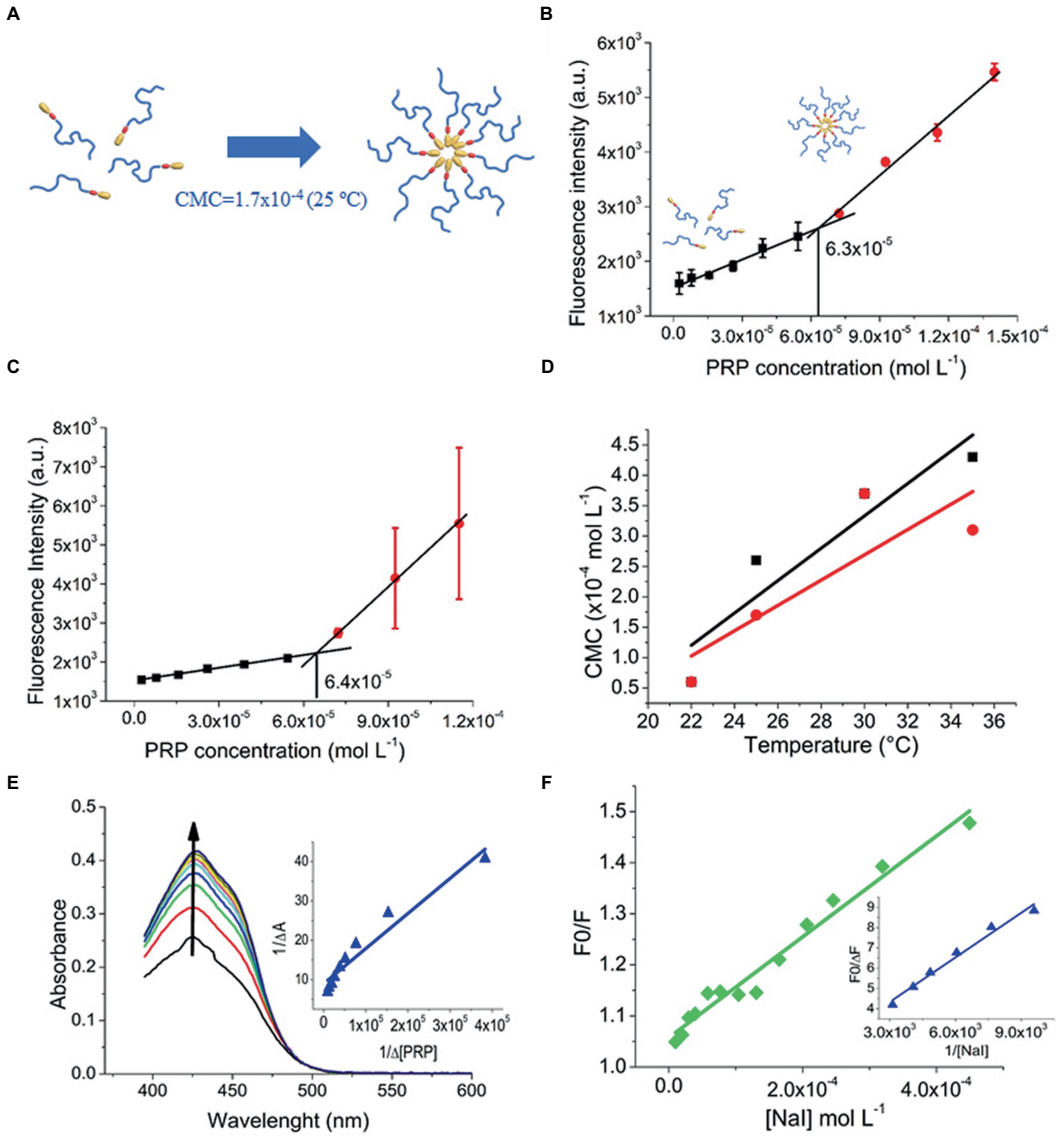
**(A)** Micelle formation at 25°C. **(B)** CMC of PRP at 22°C in the presence of pyrene. **(C)** CMC of PRP at 22°C in the presence of CUR. **(D)** Linear fitting of CMC versus temperature: PRP (black squares); CUR-loaded PRP (red circles). **(E)** Ks of PRP to CUR, the arrow indicates an increase in absorption spectra. **(F)** Fluorescence quenching of free CUR (green diamonds) and CUR-loaded PRP micelles (blue triangles).

As micellization is dynamic and concentration-and temperature-dependent, it is essential to determine the boundary concentration that triggers the transition from monomers to micelles (Figure 3A), called CMC, at different temperatures. In an aqueous PRP colloidal solution at 22°C, the fluorescence intensity of pyrene only slightly increased with a PRP concentration up to approximately 6.3 × 10^−5^ mol/L (Figure 3B). This indicated poor solubilization of pyrene under these conditions. Conversely, the increase of fluorescence intensified above 5 × 10^−5^ mol/L of PRP, showing that pyrene started to be efficiently solubilized. This indicated the presence of PRP micelles above 5 × 10^−5^ mol/L. According to PRP below and above this concentration, the distinct fluorescence behavior was linearly fitted, as illustrated in Figures 3B,C. The intersection point of the two curves was accepted as the CMC value (Alexandridis et al., 1994), and it was equal to 6.3 × 10^−5^ mol/L. Supplementary Table S4 describes the CMC values for 25, 30, and 35°C, and the supplementary material (Supplementary Figure S1) shows its fluorescence. In Figure 3D, the CMC increased as the temperature increased (in a non-linear way), indicating that micellization was exothermic at the temperature range evaluated.

The CMC of the CUR-loaded PRP was estimated with the same CUR loaded as a probe, preventing the potential effects of external probes on CMC values. Supplementary Table S4 describes these values. Below 30°C, the presence of CUR seems to favor micelle formation, and micellization tends to be unfavorable in the presence of CUR above 30°C (Supplementary Figure S2).

As a comparative study, the CMC values of F127 and P123 were determined at 25°C with and without CUR. In the case of F127, their CMC values were higher [5.8 (±2.8) × 10^−4^ mol/L and 5.7 (±1.3) × 10^−4^ mol/L, with and without CUR, respectively] than those of PRP, indicating that micelle formation was easier for PRP than F127 (Supplementary Figure S3). These findings also showed the higher hydrophobicity of PRP than F127 at that temperature.

For P123 (Supplementary Figure S4), the CMC values were relatively low [1.1 (±6.1) × 10^−6^ mol/L and 7.2 (±2) × 10^−6^ mol/L, without and with CUR, respectively] compared to those of PRP, showing that PRP was less hydrophobic than P123 at this temperature. It is worth noting that at 37°C (physiological temperature), the F127 and P123 micelles were formed in all the concentration ranges used (Sahu et al., 2011), which hinders the possibility of comparative studies with PRP.

#### 3.1.3. Determination of the equilibrium constant (K_S_)

Motivated by the effect of CUR on the formation of PRP micelles that suggested a favored interaction between CUR and PRP micelles, the Ks was determined. The K_S_ is used to quantify the interaction between receptor and ligand molecules and evaluate the complexation process (Boruah et al., 2012). Initially, the absorption spectrum of free CUR was performed (Supplementary Figure S5), which denoted peak at 425 nm. Figure 3E depicts the variation in the absorption spectrum of CUR upon titration with the PRP. Absorbance at 425 nm showed high-intensity variations in PRP addition without dislocating the absorption spectra, and such a variation suggested a favorable partition of CUR into PRP micelles. Figure 3E shows the theoretical fitting of the variation of CUR absorbance at 425 nm, according to PRP concentration. The Ks value for PRP micelles was 1.00 × 10^5^ M^−1^ (Supplementary Table S3), showing a favorable CUR interaction with PRP micelles. This is important to guarantee a high loading of CUR into PRP micelles and prevent early releases before the light-trigger step. The Ks for CUR in F127 and P123 micelles were also provided for comparison (Supplementary Figure S6); Supplementary Table S3 shows these values. The data showed that the Ks of CUR in PRP was lower than in pluronics, especially in P123 micelles.

#### 3.1.4. Fluorescence quenching

Fluorescence quenching studies may provide information related to CUR in PRP micelles. Herein, fluorescence quenching was determined by iodide titration (NaI) as a quencher agent of CUR. For free CUR, the Stern-Volmer plot was linear (Figure 3F) and the K_SV_ value was estimated at 986 l/mol (Supplementary Table S3). Besides the higher Ks value, P123 micelles showed similar behavior to water with a roughly near K_SV_ value of 797 l/mol (Supplementary Table S3).

In contrast to water and P123 micelles, the Stern-Volmer plot did not show linearity for PRP and F127 micelles. This might indicate a more heterogeneous distribution of CUR inside such micelles, in which just a fraction of CUR is accessible to iodide ions (Calori and Tedesco, 2016). In this case, a modified Stern-Volmer equation was used to consider an accessible fraction for fluorophore (Kumar et al., 2016). The F0/∆F of each CUR-loaded micelle was plotted against the inverse of iodide concentration (Figure 3F for PRP and Supplementary Figure S7 for F127 and P123 micelles). For PRP micelles, fluorescence quenching was 506 M^−1^ (Supplementary Table S3), suggesting a deeper localization of CUR into the core of micelles than P123, that is, far from most water access. The lowest fluorescence quenching value of 175 M^−1^ of CUR in F127 micelles (Supplementary Table S3) indicates the role of PEG length in the moiety protection of CUR against the aqueous agent.

#### 3.1.5. Polymer photo-response and CUR photo-triggered release

Considering that PRP micelles can load and protect CUR in its hydrophobic core away from the surrounding medium, the efficiency of the photo-triggered release of CUR by the photodegradation processes of the PRP backbone must be evaluated. Such release would decrease the absorption intensity of CUR, which would be used to assess its release rate. The PRP photodegradation was evaluated spectrophotometrically after UV light irradiation with a wavelength of 355 nm under different light fluence.

**Figure 4:**
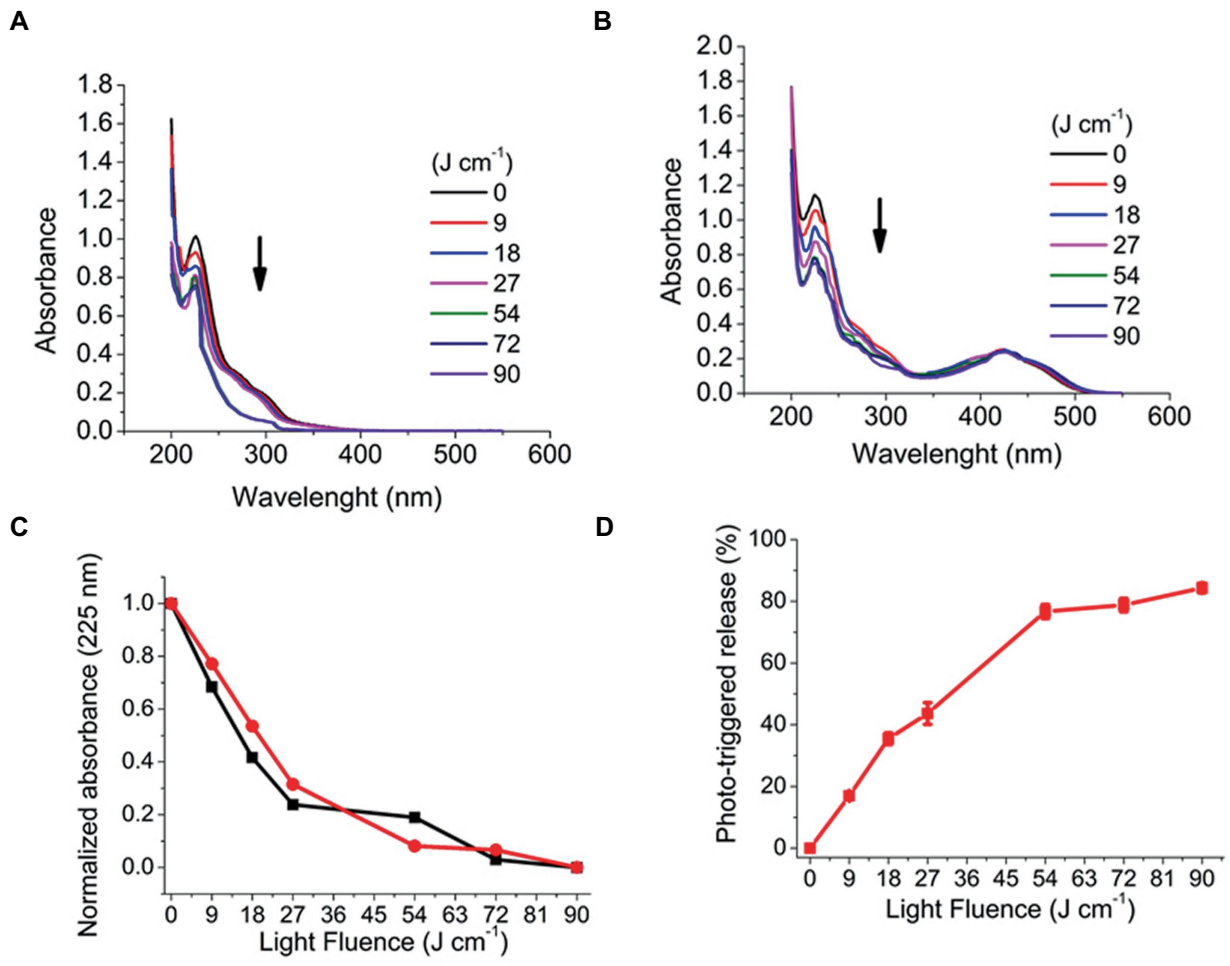
Photodegradation of PRP after irradiation with a UV laser light (355 nm, 25 mW/cm, from 6 to 60 min, corresponding to fluences of 9 to 90 J/cm). **(A)** Without CUR. **(B)** With CUR, arrows indicate decreased absorption spectra. **(C)** Normalized absorbance of photodegradation of PRP with CUR (black squares) and without CUR (red circles). **(D)** CUR release from PRP micelles.

Figure 4 shows the absorbance variation, meaning the photodegradation of PRP micelles under UV light irradiation. The CUR-free PRP (Figure 4A) showed an absorbance reduction at 225 nm from 1.00 (±0.02) to 0.76 (±0.03) after 90 J/cm^2^ of irradiation (ΔA = 0.24). In turn, CUR-loaded PRP presented a further reduction from 1.13 (±0.03) to 0.69 (±0.05) under the same light fluence (ΔA = 0.44) (Figure 4B). The normalized absorbance decrease did not show significant differences between the PRP photodegradation rates in the presence and absence of CUR (Figure 4C). After PRP photodegradation, the photo-triggered CUR release was evaluated with 0.057% w/v of PRP. The percentage of photo-released CUR was 84.3 (±4.7)% (Figure 4D) after 90 J/cm^2^ of UV light irradiation.

For CUR-loaded F127 and P123 micelles, a release of >80% was only obtained after more than 30 h in a dialysis membrane assay (Supplementary Figure S8).

### 3.2. Cytotoxicity assay

**Figure 5:**
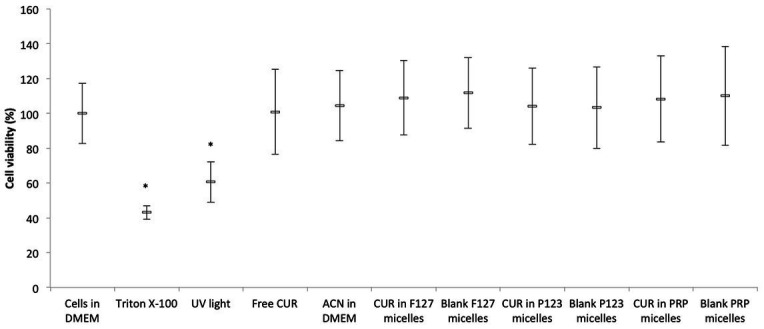
Mean values (%) of MTT after incubating NOK cells with CUR-loaded micelles. Error bars: standard deviation (*n* = 12). Asterisks (*) denote significant differences (*p* < 0.05) among the groups. The data analyzed by ANOVA/Welch and Games-Howell post-hoc tests.

The positive control group showed significant (*p* < 0.001) reduction of the absorbance values compared with the other groups. UV light also resulted in significant (*p* ≤ 0.003) diminution of the cell metabolism, although the reduction observed in the positive control group (57%) was higher than that caused by UV light (39%). Conversely, all CUR-loaded or CUR-free micelles and CUR solvents did not show significant differences (*p* ≥ 0.865) with each other and compared with the negative control group (Figure 5).

### 3.3. Antimicrobial assays

#### 3.3.1. Photoinactivation of planktonic cultures

**Figure 6:**
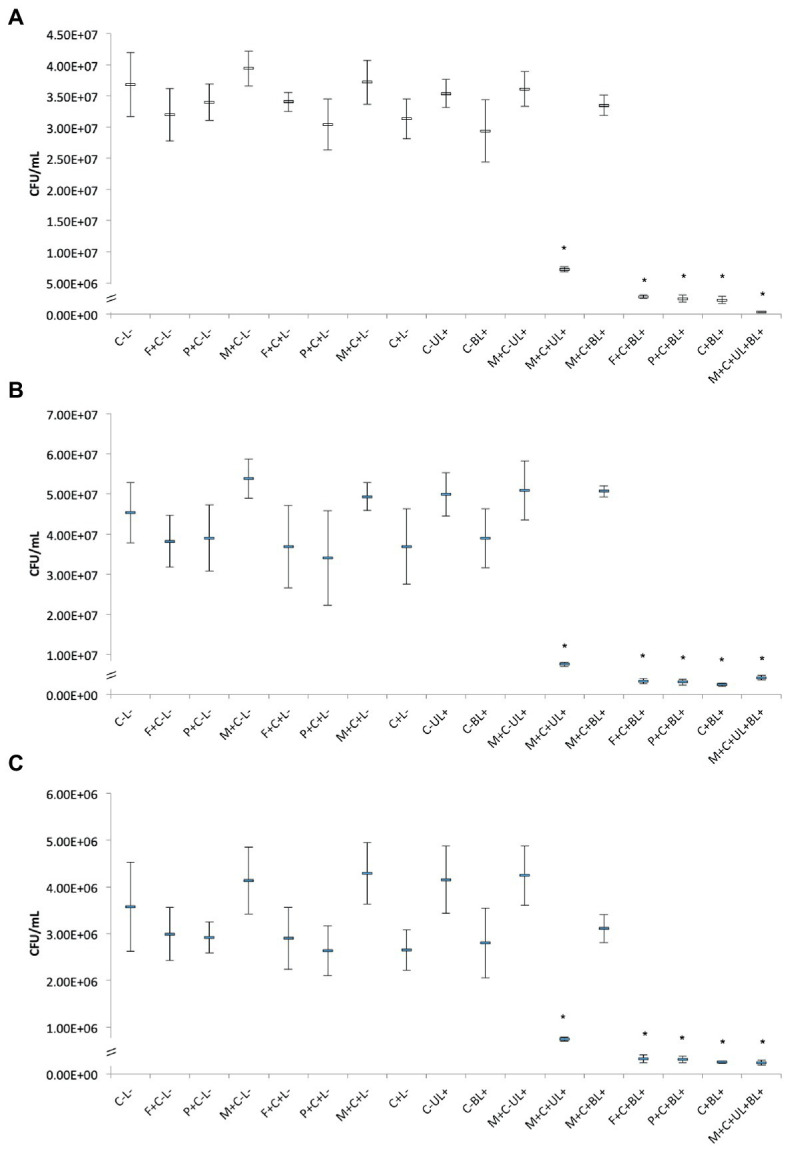
Mean CFU/mL values of planktonic cultures of MRSA **(A)**, *P. aeruginosa* **(B)**, and *C. albicans* **(C)** obtained in each group of study. Error bars: standard deviation (*n* = 12). Asterisks (*) denote significant difference (*p* < 0.05) compared with the control (C-L-). The data was analyzed by ANOVA/Welch and Games-Howell post-hoc tests. Groups are described in the Supplementary Chart S1 of Supplementary Information.

The antimicrobial effect of CUR-loaded PRP micelles was compared with that of CUR-loaded F127 and P123 micelles and free CUR. For all the three species evaluated in planktonic cultures (Figure 6), the UV irradiation alone (C-UL+) did not show significant difference (*p* > 0.05) compared with the control group (C-L-), i.e., UV light did not show antimicrobial activity.

For planktonic MRSA, the association of CUR-loaded PRP micelles with UV and blue lights (M + C + UL + BL+) significantly (*p* < 0.001) decreased bacterial viability by 2 log_10_(CFU/mL) compared with the untreated control group (C-L-). The combination of free CUR (C + BL+) and CUR-loaded F127 (F + C + BL+) and P123 micelles with blue light (P + C + BL+) reduced bacterial viability by 1 log_10_(CFU/mL) compared with the untreated control and was statistically different (*p* < 0.001) from all other groups (Figure 6A).

Figure 6B shows the antimicrobial results against planktonic *P. aeruginosa*. Bacterial samples subjected to CUR-loaded PRP micelles associated with UV and blue lights (M + C + UL + BL+), conventional micelles (F + C + BL+ and P + C + BL+) and free CUR combined with blue light (C + BL+) resulted in a significant (*p* ≤ 0.048) reduction of 1 log_10_(CFU/mL) compared with the untreated control group.

For planktonic *C. albicans*, the associations of free CUR with blue light (C + BL+), CUR-loaded PRP micelle with UV and blue lights (M + C + UL + BL+) and CUR-loaded conventional micelles with blue light (F + C + BL+ and P + C + BL+) also reduced (*p* < 0.001) yeast viability by 1 log_10_(CFU/mL) compared with the untreated control group (C-L-, Figure 6C).

The present investigation also showed that irradiating CUR-loaded PRP micelles with a UV light (M + C + UL+) significantly (*p* ≤ 0.001) reduced the three microbial species evaluated by less than 1 log_10_(CFU/mL). The UV irradiation of CUR-free PRP micelles (M + C-UL+) did not reduce the viability of the three microbial species (Figures 6A–C), confirming that CUR was responsible for the antimicrobial effect after UV light irradiation.

#### 3.3.2. ROS production

**Figure 7:**
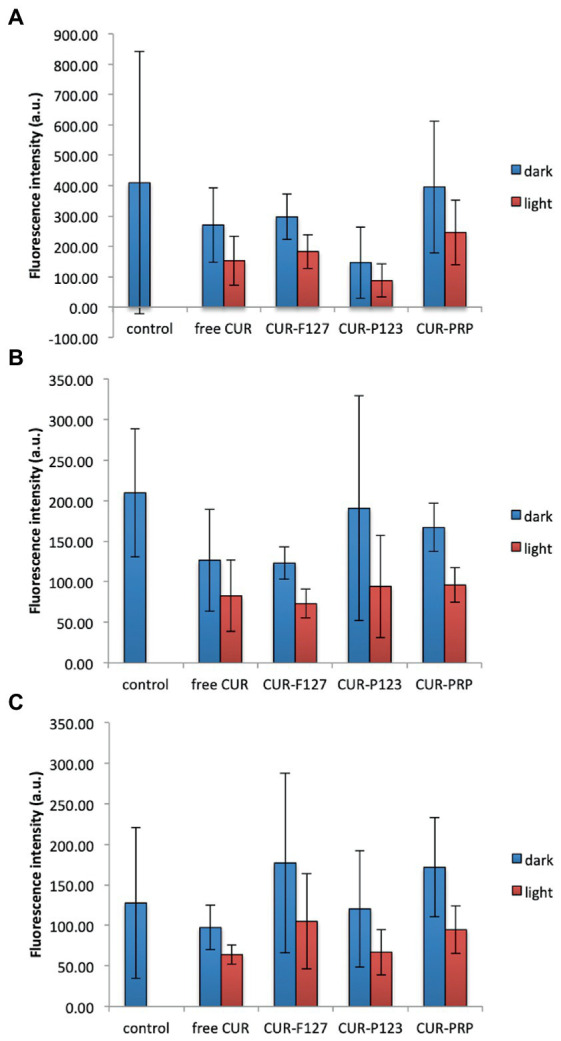
Mean values of intracellular ROS production (fluorescence intensity in arbitrary units, a.u.) of MRSA **(A)**, *P. aeruginosa* **(B)**, and *C. albicans* **(C)** after light irradiation of CUR in its free form and loaded into the micelles (F127, P123, and PRP) or incubation with the photosensitizers in the dark. Control: untreated cells. Error bars: standard deviation (*n* = 3). The data was analyzed by ANOVA/Welch and Games-Howell post-hoc tests.

For the three microbial species evaluated, the statistical analysis did not demonstrate increase in the ROS production after light irradiation compared with the microbial samples treated only with the photosensitizers (free CUR and CUR loaded in F127, P123, and PRP micelles) in the dark (*p* ≥ 0.141) (Supplementary Figure S9). Unexpectedly, the untreated controls (microbial samples incubated only with DCFHDA) and samples incubated with the PS in the dark showed higher fluorescence intensity values than those submitted to light. Considering that the high values observed for the samples in the dark might be attributed to the presence of the PS, a new series of experiments (*n* = 3) was performed centrifuging (10,000 rpm for 5 min) and washing the microbial cell suspensions with PBS after incubating them with the DCFHDA and after the incubation with the PS in order to detect only the intracellular ROS and exclude a possible interference of extracellular PS. Nonetheless, similar results were observed: samples in the dark showed higher mean values than those submitted to light without significant difference (Figure 7).

#### 3.3.3. Metabolic activity of biofilms

**Figure 8:**
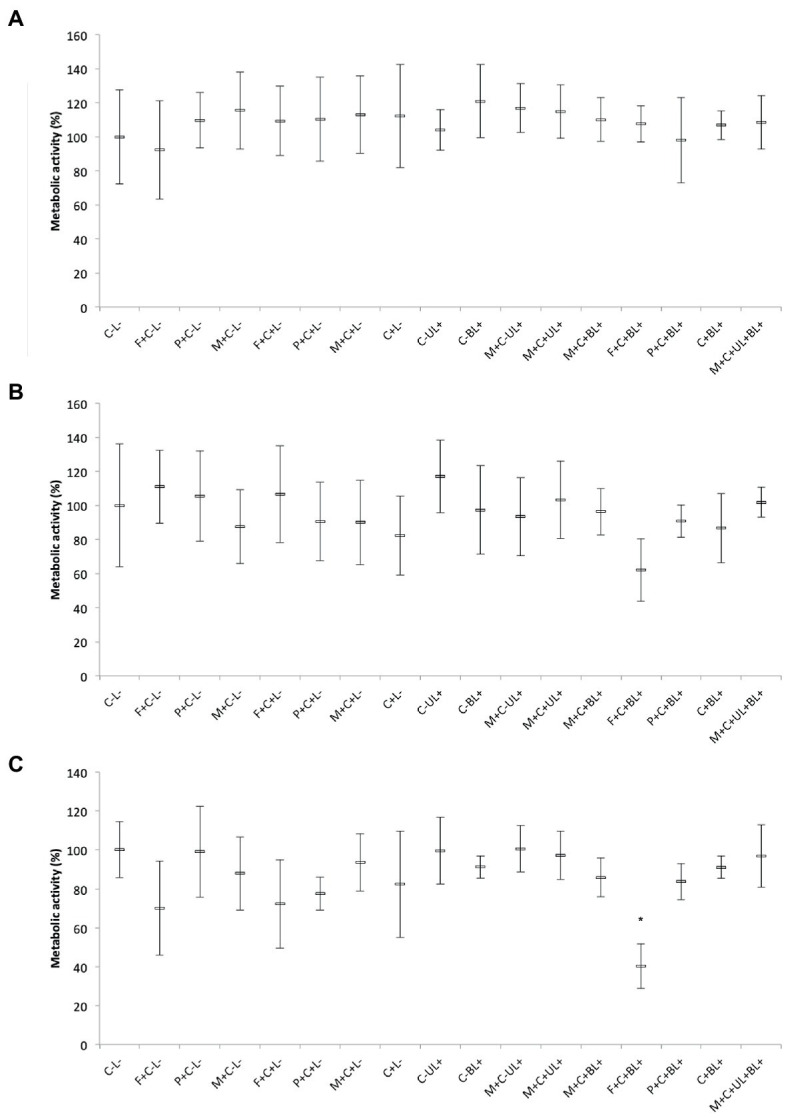
Mean values of metabolic activity (%) of MRSA **(A)**, *P. aeruginosa* **(B)**, and *C. albicans* **(C)** biofilms obtained in each group of study. Error bars: standard deviation (*n* = 12). Asterisks (*) denote significant differences (*p* < 0.05) compared with the control (C-L-). The data was analyzed by ANOVA/Welch and Games-Howell post-hoc tests. Groups are described in the Supplementary Chart S1 of Supplementary Information.

The results obtained for bacteria, MRSA and *P. aeruginosa*, showed that the combination of PS and light did not significantly affect (*p* ≥ 0.196) the metabolic activity of biofilms (Figures 8A,B). Although there were no significant differences among the groups, the biofilm of *P. aeruginosa* subjected to CUR-loaded F127 micelles combined with blue light (F + C + BL+) showed the lowest mean value of the metabolic activity, 37% lower than the mean value of the untreated control group (C-L-, Figure 8B).

Conversely, for *C. albicans* biofilms, CUR-loaded F127 micelles associated with blue light (F + C + BL+) reduced the metabolic activity by 59% compared with the untreated control group and showed a significant difference (*p* ≤ 0.028) from the other groups, except the one treated only with CUR-free F127 micelles (F + C-L-, *p* = 0.07). Therefore, our results showed that only CUR-loaded F127 micelles resulted in biofilm photoinactivation (Figure 8C).

#### 3.3.4. Biofilm biomass

**Figure 9:**
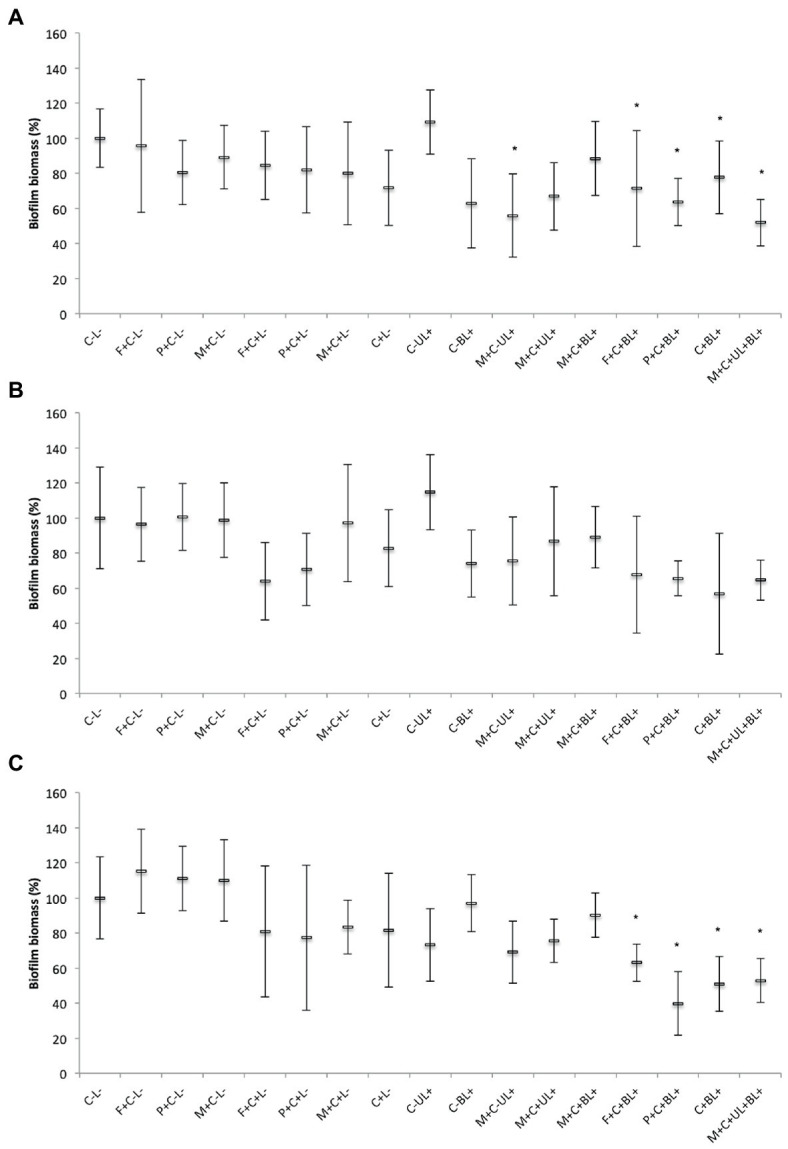
Mean values of biofilm biomass (%) of MRSA **(A)**, *P. aeruginosa* **(B)**, and *C. albicans* **(C)** obtained in each group of study. Error bars: standard deviation (*n* = 12). Asterisks (*) denote significant differences (*p* < 0.05) compared with the control (C-L-). The data was analyzed by ANOVA/Welch and Games-Howell post-hoc tests. Groups are described in the Supplementary Chart S1 of Supplementary Information.

For single biofilms of MRSA and *C. albicans*, CUR-loaded PRP micelles combined with UV and blue lights (M + C + UL + BL+) reduced (*p* < 0.001) the biofilm biomass by 48 and 47%, respectively, compared with the untreated control group (C-L-, Figures 9A,C). Moreover, for the Gram-positive bacterium and yeast, associating free CUR (C + BL+) and CUR-loaded F127 and P123 micelles with blue light (F + C + BL+ and P + C + BL+) decreased the biofilm biomass up to 36% (*p* ≤ 0.001) and 30% (*p* ≤ 0.010), respectively, compared with the untreated control group (C-L-).

For *P. aeruginosa*, there was no significant difference (*p* ≥ 0.067) in the biofilm biomass between the groups subjected to PS and light (F + C + BL+, P + C + BL+, C + BL+, and M + C + UL + BL+) and the untreated control group (C-L-, Figure 9B). Therefore, our results from biofilm biomass showed that MRSA and *C. albicans* were more susceptible to the combination of free CUR and CUR-loaded micelles with light than *P. aeruginosa*.

#### 3.3.5. Ps uptake by the biofilms

**Figure 10:**
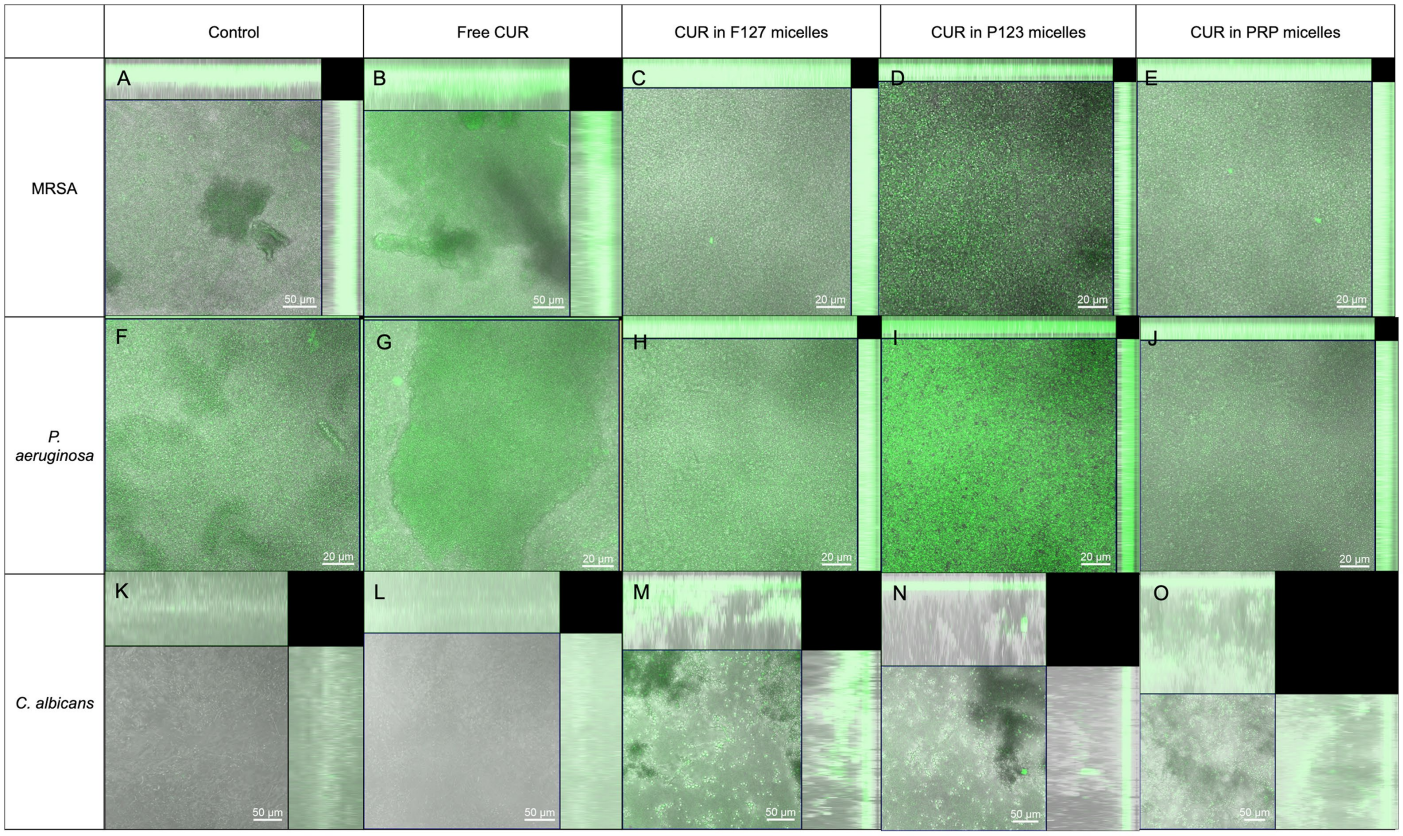
Confocal microscopy images showing the fluorescence of biofilms incubated only with the culture media [controls **(A,F,K)** or CUR in its free form **(B,G,L)**] or loaded into the micelles (F127 in **C,H,M**; P123 in **D,I,N**; and PRP in **E,J,O**) by the biofilms of MRSA **(A–E)**, *P. aeruginosa* **(F–J)**, and *C. albicans* **(K–O)**. Images are overlap of bright field and fluorescence mode.

Figure 10 shows the confocal images of each biofilm grown with the different PS (free CUR, CUR-loaded PRP, F127, and P123 micelles, and untreated controls with PBS). The biofilm’s thickness and the fluorescence intensity values obtained for each image are shown in Supplementary Table S5. The untreated control biofilm of *C. albicans* showed autofluorescence higher than that grown with free CUR and the untreated control biofilm of *P. aeruginosa* demonstrated autofluorescence higher than those grown with free CUR and CUR-loaded PRP micelles, suggesting that the uptake was weak for these CUR-treated samples. Conversely, the highest fluorescence intensities were observed for MRSA biofilm treated with free CUR, *P. aeruginosa* biofilm treated with CUR in P123 micelles, and *C. albicans* biofilm treated with F127 micelles. The other samples showed fluorescence intensity values higher than the untreated controls. In addition, the highest thicknesses were observed for *C. albicans* biofilms due to the presence of long filamentous forms (hyphae and pseudo-hyphae).

## 4. Discussion

CUR in nanocarries has been researched as an alternative for treating microbial infections and as PS in aPDT (Dovigo et al., 2011a,b; Quishida et al., 2016; Trigo-Gutierrez et al., 2021). Stimuli-responsive nanosystems can improve the drug delivery at the target of interest in a spatial-and temporal-controlled fashion, which would be promising for topical therapies against biofilm-related infections, and light is an exogenous stimulus that can also be employed in aPDT. To the best of our knowledge, no other study has evaluated photo-responsive micelles for CUR release in aPDT. Thus, in this study, we designed a PRP to carry CUR as a photo-responsive micelle to be used as a PS in aPDT. Moreover, we compared the aPDT mediated by CUR-loaded in photo responsive micelles with Pluronic F127 and P123 micelles, as well as free CUR.

The PRP was formed by three main segments: PEG as hydrophilic segment that enhances the anti-protein absorption on micelle surface (Shi et al., 2021). PEG has been commonly explored to improve the stability and biocompatibility of several nanomaterials initially insoluble in water (Veronese and Pasut, 2005). Another segment of PRP was formed by BNA as a photo-responsive segment, which can be easily broken to release the drug (Hou et al., 2020), and the third segment was PAA as the hydrophobic segment. A similar approached was employed by other studies, in which nitrobenzyl was conjugated to polymers to synthesize a UV-responsive nanocarrier for pesticide (Ye et al., 2015; Ding et al., 2016).

The zeta potential of PRP micelles was slightly negative and can be attributed to the electrical surface of the PEG segment of PRP (Shi et al., 2021). A high surface charge is important for the stability of the nanoformulation promoted by electrostatic repulsion between the nanoparticles (Sharma et al., 2022). Therefore, the surface charge of the micelles observed in our study could be considered a limitation and, for this reason, all biological assays were performed immediately after the synthesis of the micelles. Moreover, cationic nanosystems are more effective against microorganisms and biofilms, because they are attracted by the negative charge of the cell wall and the biofilm matrix (Fulaz et al., 2019). Despite their efficacy, cationic nanoparticles can also demonstrate cytotoxic effect against mammalian cells (Trigo Gutierrez et al., 2017). Nonetheless, the interaction between nanoparticles and macromolecules can alter their surface properties, resulting in the formation of the protein corona, which is often reported in blood plasma, but not described in biofilms yet (Fulaz et al., 2019). Besides the electrostactic interaction, hydrophobic and steric interactions have been also described as important physicochemical factors that dictate the biofilm-nanoparticle interaction (Fulaz et al., 2019).

The PRP showed higher EE% value than those observed for pluronic micelles that could be related to the aromatic segment in PRP structure, as it was reported in a previous study (Naksuriya et al., 2015). It is worth noting that the drug:polymer ratio highly modulates EE%. A ratio variation from 1:5 to 1:50 promoted a difference from 34.33 to 95.57% on the EE% of CUR in F127 micelles (Sahu et al., 2011).

The CMC at 25°C demonstrated higher hydrophobicity of PRP than F127. This feature comes from the hydrophobic BNA-PAA moiety of the PRP, which is responsible for the self-assembly into micelles in water and free energy reduction. Subsequently, a low polymer concentration is required for micellization (Akbar et al., 2018). Moreover, the presence of *o*-nitrobenzyl esters increased the hydrophobicity of the hydrophobic chain (Liu et al., 2015), as reported in a previous study (Ye et al., 2015) that showed a CMC variation from 0.057 mg mL^−1^ to 0.019 mg mL^−1^ in the synthesis of 2-nitrobenzyl succinate-carboxymethyl chitosan micelles.

The interaction between receptor and ligand molecules was determined by the K_S_, which showed lower value for CUR in PRP than in pluronics. The difference suggests that the hydrophobic/hydrophilic balance in the microenvironment could modulate the partition of CUR. Considering that the asymmetry of the PRP provides a gradient of hydrophobicity from the shell to the core of micelles, forming various sites of location for the loaded CUR, the preferred binding site of CUR must be evaluated.

The fluorescence quenching studies using water-soluble quenchers, including iodide ions (Lakowicz, 2006) may provide information about the availability of PS to the external environment, such as blood plasma proteins, which could compete with CUR delivery by decreasing the load of PRP micelles. The findings showed that CUR is not partitioned to the core of the P123 micelle, but is located in a more accessible site for iodide ions such as the PEG shell. The short PEG of PRP might further contribute to the access of iodide ions, resulting in the high K_SV_ value obtained. The PEG shell works as a steric barrier, protecting CUR from biodegradation and potential interactions with serum proteins (Shi et al., 2021). The lowest fluorescence quenching value of 175 M^−1^ of CUR in F127 micelles indicated the role of PEG length in the moiety protection of CUR against the aqueous agent. These findings showed the efficacy of PRP and PEG shell as protectors of CUR, at least *in vitro*.

The PRP is an amphiphilic molecule composed of a hydrophilic part of PEG that forms the shell of micelles and a hydrophobic part comprising phenoxyacetic acid and *o*-nitrobenzyl, which constitute the micellar core. The photocleavage process would break the connection between the hydrophobic group and the hydrophilic PEG, disrupting the micelle and releasing CUR to the aqueous medium. The photodegradation of polymers using UV light (355 nm) can be explained by the cleavage of the PEG-o-nitrobenzyl ester group (Holmes and Jones, 1995). The photocleavege observed here was similar to other studies (Liu et al., 2015; Ding et al., 2016), but without the formation of another peak or shoulder after photocleavage, which was also observed in other studies (Cao et al., 2017; Zhao C. et al., 2020).

The photo-triggered CUR release observed here was lower than those described in previous reports (Ye et al., 2015; Ding et al., 2016) that showed photo-release values of 99.5% (Ding et al., 2016) and 96.9% (Ye et al., 2015) for a pesticide conjugated to UV-responsive micelles. These higher values are partly because the pesticide is chemically conjugated to the PRP, compared to the physical loading of CUR in our investigation. Another study using mesoporous hollow titania nanoparticles reported up to 96% of CUR release after UV light irradiation (Mai et al., 2021). This higher release could be attributed to the mesoporous feature of the nanocarrier, which may ease the CUR release.

After the micelles’ characterization we evaluated their cytotoxicity. The results demonstrated that PRP, F127, and P123 micelles did not show cytotoxicity *in vitro*, which motivates further *in vivo* studies. These findings can be at least partially attributed to the biocompatibility of the PEG chain, approved by the Food and Drug Administration for clinical use (Deshmukh et al., 2017; Zhao X. et al., 2020). Moreover, the conjugation of BNA and PAA to PEG did not result in cytotoxicity. Conversely, UV light was toxic to mammal cells, but this result should be seen with caution because cells cultured in monolayers do not reproduce dynamic and complex environments of tissue. Therefore, more appropriate models such as 3D cell cultures are required to evaluate toxicity and biocompatibility that may be observed *in vivo*. Moreover, the cytotoxicity of UVA light (320–400 nm) to cells is higher with repeated irradiations with short intervals than with continuous single dose (Merwald et al., 2005). Another study also demonstrated that aPDT mediated by CUR was toxic to human gingival fibroblast cells cultured as monolayers (Pourhajibagher et al., 2016).

Considering the lack of cytotoxicity observed *in vitro* and the hypothesis that a photo-controlled CUR release would be effective for photoinactivation of Gram-positive (MRSA) and Gram-negative (*P. aeruginosa*) bacteria and yeast (*C. albicans*), we used CUR-loaded PRP micelles as a PS for aPDT. Importantly, UVA (369 nm) irradiation alone used for CUR photorelease did not result in antimicrobial effect against the species evaluated, which agrees with previous studies (Wang et al., 2019; Boluki et al., 2020). UVA light reduced the viability of resistant *Acinetobacter baumannii* (19.2 J/cm^2^) (Boluki et al., 2020) and *Escherichia coli* (370 J/m^3^) (Wang et al., 2019) by only 3.01% and 0.5 lg, respectively. Moreover, UVD (100–200 nm) and UVC (200–275 nm) showed higher antibacterial effect than UVA (Wang et al., 2019). Despite the risks of UVA light on mammalian cells (McMillan et al., 2008), a recent systematic review demonstrated that UVA and UVC lights were effective for treating superficial human infections, especially microbial keratitis, with adverse effects similar to the standard treatment or placebo group (Marasini et al., 2021). Another review about the effect of UV light on skin (Matsumura and Ananthaswamy, 2004) showed that UVA light is more associated with photoaging, while UVB (280–315 nm) is more mutagenic, and both UV lights are used as therapeutic modalities for human skin diseases, such as psoriasis, vitiligo, atopic dermatitis, and cutaneous T-cell lymphoma. However, acute (erythema) and chronic side effects (carcinogenesis) have been observed; the chronic side effects are related to the number of treatments and cumulative UV doses. The maximum absorbance of UV light by DNA varies from 245 to 290 nm, so UVC irradiation has more potential to cause DNA damage, although microbial cells are more susceptible to UVC irradiation than mammalian cells, which are equipped with DNA repairing systems (Matsumura and Ananthaswamy, 2004; Yin et al., 2013).

After UV and blue light irradiation of CUR-loaded PRP micelles, the viability of planktonic MRSA, *P. aeruginosa*, and *C. albicans* decreased by 1–2 log_10_ (CFU/mL) and the susceptibility range was MRSA > *P. aeruginosa* = *C. albicans*. In our previous study (Trigo Gutierrez et al., 2017), aPDT mediated by CUR loaded in anionic, polymeric nanoparticles resulted in reduction of MRSA and *C. albicans* by 6.47 and 0.78 log_10_, respectively. The higher reduction observed for MRSA may be ascribed to the higher concentration of CUR loaded in the nanoparticles (260 μM). Polyvinylpyrrolidone complexes with 5 μM CUR resulted in the photoinactivation of *S. aureus* from 1 to 6 log_10_, according to the incubation time used before irradiation (Winter et al., 2013). Increased incubation times from 5 to 25 min with 5 μM CUR reduced photoinactivation, while complete photoinactivation occurred at longer incubation times with 50 μM CUR (Winter et al., 2013). Different incubation times and CUR concentrations loaded in the micelles were not evaluated, which would be considered a limitation of our study. However, previous studies from our group demonstrated that higher incubation time of CUR did not increase photoinactivation of *Candida* spp. (Andrade et al., 2013). Our previous study (Trigo Gutierrez et al., 2017) also demonstrated that washing cells incubated with the PS reduced the photoinactivation, because the PS on the bulk solution and not bound to the microbial cells also participate in the photoinactivation (Dahl et al., 1989; Dovigo et al., 2011a,b). In our previous study, we observed that washing the PS abolished the photoinactivation of *C. albicans* (Trigo Gutierrez et al., 2017). Singlet oxygen produced by aPDT diffuses approximately 100 nm and has a lifetime of <0.4 μs (Soukos and Goodson, 2011), thus the ROS generated by the PS not bound to but surrounding the cells may play a role in photoinactivation. Moreover, washing the PS reduces its concentration, which may affect the photodynamic efficacy.

As expected, the reduction in *P. aeruginosa* was lower than that of MRSA because Gram-negative species are less susceptible to photoinactivation (Huang et al., 2012) due to the presence of an outer membrane on their cell walls that is not present in Gram-positive bacteria. For an effective photoinactivation of Gram-negative species, cationic PS and cell wall permeabilizing agents have been used (Huang et al., 2012). In studies with planktonic cultures of *P. aeruginosa*, contradictory results have been reported. Spermine micelles loaded with 50 and 100 nM CUR combined with 18 and 30 J/cm^2^ of blue laser light resulted in bacterial photoinactivation, which was evaluated by absorbance at 600 nm (Rupel et al., 2020). In contrast, free CUR at 200 μM combined with 6, 18, and 30 J/cm^2^ was ineffective in the photoinactivation of *P. aeruginosa* (Rupel et al., 2020). Another study (Zhou et al., 2021) showed a 1-log reduction of planktonic *P. aeruginosa* after aPDT mediated by 120 μM CUR, combined with 10 J/cm^2^ of blue light. Furthermore, combining 100 μM CUR with the cationic polypeptide polymyxin B enhanced the effect of aPDT, decreasing 2 log (Zhou et al., 2021). The CUR-mediated aPDT caused membrane depolarization, morphological deformation, and DNA and protein damage in the bacterium (Zhou et al., 2021). Therefore, our results corroborate those reported by others (Rupel et al., 2020; Zhou et al., 2021).

The 1-log_10_ reduction of *C. albicans* after UV and blue-light irradiation of CUR-loaded PRP micelles agreed with our previous study (Trigo Gutierrez et al., 2017), which reported 0.79 log_10_ reduction after aPDT mediated by CUR-loaded in polymeric nanoparticles. On the other hand, another study (Schamberger and Plaetzer, 2021) reported that aPDT mediated by free CUR at 50 μM decreased the viability of planktonic *C. albicans* by 3 log. Furthermore, a water-soluble positively-charged CUR derivative at 100 μM increased the photodynamic effect, reducing fungal viability by 6 log (Schamberger and Plaetzer, 2021). A complex formed by CUR and sophorolipid (a surfactant from non-pathogenic yeast) at 100 μg/mL inhibited the growth of *C. albicans* by more than 70%, measured with an optical density at 595 nm, while CUR alone at 9.37 μg/mL reduced fungal growth by 47% (Rajasekar et al., 2021). Therefore, the higher photoinactivation of *C. albicans* observed in other studies (Rajasekar et al., 2021; Schamberger and Plaetzer, 2021) may be attributed to higher CUR concentrations and the positive charge of the PS.

Our study also demonstrated that aPDT did not increase the intracellular ROS production. This result does not corroborate those found by others (Parasuraman et al., 2019; Muehler et al., 2020), which reported increased ROS levels after aPDT. The lack of significant difference in our investigation may be due to the short lifetime of ROS. Singlet oxygen produced by aPDT has short lifetime and limited diffusion, and the lifetime of PS at the triplet state ranges from microseconds to milliseconds (Soukos and Goodson, 2011). Considering that the ROS produced by unbound PS may target the cell membrane, measuring the ROS production during illumination and in the extracellular milieu may be more accurate. Therefore, we also measured the ROS production after centrifuging and washing the microbial cells incubated with the DCFHDA and the PS, but similar results (no significant difference among the groups) were observed. Moreover, the untreated controls (microbial samples incubated with DCFHDA) and samples incubated with the PS in the dark showed higher ROS production than those submitted to aPDT. A possible explanation of these findings may be the reduction of cell viability after aPDT. Since DCFHDA is a probe for intracellular ROS, the 1–2 log reduction (more than 90% of reduction) of cell viability caused by aPDT may consequently cause the reduction in the ROS detection. Probably, intracellular ROS detection using DCFHDA would be more appropriate for living cells. Another study also reported high ROS production in untreated microbial cells, although aPDT resulted in a significant increase in the ROS generation (Muehler et al., 2020). More recently, it was proposed an oxygen-independent antimicrobial photoinactivation (type III photochemical mechanism), in which photoinduced electron transfer produces reactive inorganic radicals, leading to microbial killing (Hamblin and Abrahamse, 2020). This hypothesis may explain our photoinactivation findings without increased ROS production.

For biofilms, any PS combined with light did not reduce the metabolic activity of MRSA and *P. aeruginosa*. This result may be also explained by low concentration of CUR loaded in the micelles and the micelles’ negative charge. Other studies verified reduction in the metabolic activity of MRSA and *P. aerugonosa* biofilms using higher concentrations of free CUR (Abdulrahman et al., 2020; Ribeiro et al., 2022). For *C. albicans* biofilm, only CUR-loaded F127 micelles associated with blue light reduced the metabolic activity by 59%. This result agrees with that from another study, in which aPDT mediated by 5 μM CUR and 37.5 J/cm^2^ of light decreased the metabolic activity of the *C. albicans* biofilm by up to 29% (Dovigo et al., 2011a).

Biofilms are complex microorganism communities embedded in a self-produced polymeric matrix. Although the combination of PS and light did not reduce the metabolic activity of the MRSA biofilm, the reduction of biofilm biomass observed here may be attributed to a potential effect of such combination on the biofilm matrix. The results of biofilm biomass agree with those from other studies, which used higher CUR concentrations against biofilms. A previous study showed that 256 μg/mL of CUR decreased the viability of a 48-h-old MRSA biofilm by 54% (de Andrade Neto et al., 2021). In another study (Akhtar and Khan, 2021), 2,500 μg/mL CUR alone reduced by 28% the biofilm biomass of vancomycin-resistant *S. aureus*, while CUR combined with 20 J/cm^2^ of light reduced biofilm biomass by 80%. For *C. albicans*, 9.37 μg/mL CUR in a sophorolipid nanocomplex decreased biofilm biomass when added before and after the adhesion phase of fungal cells (Rajasekar et al., 2021). Therefore, the reduction of biofilm biomass observed in our study for MRSA and *C. albicans* is promising, considering that a low concentration of CUR was used. The images observed in CLSM demonstrated uptake of the PS by MRSA and *C. albicans* biofilms, which corroborated with the reduction in biomass observed.

On the other hand, no reduction of *P.* aeruginosa biofilm biomass was observed. This result may be attributed to the low concentration of CUR-loaded micelles used against the bacterial biofilm. Although the CLSM images demonstrated intense fluorescence of CUR-loaded PRP micelles in *P. aeruginosa* biofilm, only some cell clusters incorporated the PS, which probably was not enough to reduce the biofilm metabolism and biomass. A higher concentration of CUR (200 μg/mL) combined with 20 μg/mL of silver nanoparticles (AgNP) irradiated at 10 J/cm^2^ inhibited the biofilm formation of *P. aeruginosa* by 85% (Ghasemi et al., 2021). Conversely, AgNP irradiation and aPDT mediated only by CUR decreased biofilm formation by 60 and 50%, respectively (Ghasemi et al., 2021). In another study (Abdulrahman et al., 2020), aPDT mediated by 6.75 mM CUR and light at 5 and 10 J/cm^2^ inhibited the biofilm formation of *P. aeruginosa* by 40 and 70%, respectively. Therefore, our results from biofilm biomass demonstrated that the association of free CUR and CUR-loaded micelles with light was more effective against the biofilm biomass of MRSA and *C. albicans* than that of *P. aeruginosa*. The lower susceptibility of *P. aeruginosa* biofilm may be explained by its matrix composition, which is comprised of a mixture of exopolyssacharides (EPS, major fraction), extracellular DNA (eDNA) and proteins. The EPS includes alginate, mannose-rich Psl, and glucose-rich Pel (Reichhardt and Parsek, 2019). On the other hand, staphylococci produces only one main EPS, the polysaccharide intercellular adhesin (PIA) (Nguyen et al., 2020), while the EPS of *C. albicans* biofilm consist of α-mannans, 1,6-β-glucan, and 1,3-β-glucan (Zarnowski et al., 2014). Therefore, the different composition of their biofilm matrix, especially the EPS content and its complexity, may explain the different susceptibilities of the biofilms to the antimicrobial approach. Another study also observed lower susceptibility of *P. aeruginosa* biofilm to aPDT than that of *S. aureus* and *C. albicans* (Beirão et al., 2014).

As conclusion, this study designed a photo-responsive polymeric micelle for the loading and light-triggered release of CUR. The nanoscale of CUR-loaded photo-responsive micelles, its polydispersity index, and low cytotoxicity *in vitro* is a proof-of-concept of its applicability as a photo-responsive system for antimicrobial therapies. Moreover, the low CMC values of PRP guarantee its self-aggregation into micelles even at low concentrations and prevent the early release of CUR by dilution effects. The high association constant between CUR and polymeric micelles favors an efficient drug loading, and photocleavage releases CUR during light therapy. Finally photo-responsive polymeric micelles showed an antimicrobial effect against pathogenic microorganisms and biofilms, especially against Gram-positive bacterium and fungus, when associated with light. These findings approve the CUR-loaded photo-responsive micelle for antimicrobial approaches. Further studies should focus on higher CUR concentrations loaded into the micelles, cationic functionalization of light-responsive micelles, and *in vivo* evaluations using animal models of infection.

## Data availability statement

The original contributions presented in the study are included in the article/Supplementary material, further inquiries can be directed to the corresponding author.

## Author contributions

EM and JT-G: conceptualization, formal analysis, and investigation. IC, RG, and WC: methodology (photo-responsive polymer synthesis). JT-G and IC: characterization of the photo-responsive polymer and micelles. EM, JT-G and GB: cytotoxicity and microbiological assays. EM, AT, and AP: resources. JT-G, IC, GB, AT, and EM: data curation. JT-G: writing – original draft preparation. JT-G, IC, AP, AT, and EM: writing – review and editing. AT and EM: supervision. EM: project administration. AP, AT, and EM: funding acquisition. All authors contributed to the article and approved the submitted version.

## Funding

This study was supported by São Paulo Research Foundation INCT 2014/50857-8 (FAPESP, grants 2018/02513-9, 2019/21344-6, and RIDC 13/07276-1). JT-G received a doctorate scholarship from the National Council for Scientific and Technological Development - Process 142184/2018-7. IC received a FAPESP Post-Doc fellowship (2020/04507-6).

## Conflict of interest

The authors declare the competing financial interest of a patent deposited on the National Institute for Industrial Property (INPI, Brazil) on December 15, 2021, process number BR 102021 025155 7, entitled *Desenvolvimento de Micela Polimérica Fotoativada com Curcumina e suas Aplicações* (Development of a Polymeric Micelle Photoactivated with Curcumin and its Applications). There are no other competing interests.

## Publisher’s note

All claims expressed in this article are solely those of the authors and do not necessarily represent those of their affiliated organizations, or those of the publisher, the editors and the reviewers. Any product that may be evaluated in this article, or claim that may be made by its manufacturer, is not guaranteed or endorsed by the publisher.
