# Supplementary material for: Photo-responsive polymeric micelles for the light-triggered release of curcumin targeting antimicrobial activity
Source: Front Microbiol. 2023 Apr 20;14:1132781. doi: 10.3389/fmicb.2023.1132781 (PMC10157243; doi:10.3389/fmicb.2023.1132781)
Supplement: Supplementary file 1 [file Data_Sheet_1.docx]

Supplementary Material

Photo-Responsive Polymeric Micelles for the Light-Triggered Release of Curcumin Targeting Antimicrobial Activity

Jeffersson Krishan Trigo-Gutierrez, Italo Rodrigo Calori, Geovana de Oliveira Bárbara, Ana Claudia Pavarina, Renato Sonchini Gonçalves, Wilker Caetano, Antonio Claudio Tedesco, Ewerton Garcia de Oliveira Mima*

*** Correspondence:** Prof Dr Ewerton Garcia de Oliveira Mima: ewerton.mima@unesp.br

# Supplementary Data

**Chart S1.** Groups and experimental condition evaluated for each microbial species.

| **Group** | **Experimental condition** | |
| --- | --- | --- |
|  | **PS** | **Light** |
| C-L- (untreated control) | No (received PBS) | No |
| F+C-L- | F127 micelles without CUR | No |
| P+C-L- | P123 micelles without CUR | No |
| M+C-L- | PRP micelles without CUR | No |
| F+C+L- | F127 micelles with CUR | No |
| P+C+L- | P123 micelles with CUR | No |
| M+C+L- | PRP micelles with CUR | No |
| C+L- | Free CUR | No |
| C-UL+ | PBS | UV 1 hour |
| C-BL+ | PBS | Blue light 20 minutes |
| M+C-UL+ | PRP micelles without CUR | UV 1 hour |
| M+C+UL+ | PRP micelles with CUR | UV 1 hour |
| M+C+BL+ | PRP micelles with CUR | Blue light 20 minutes |
| F+C+BL+ | F127 micelles with CUR | Blue light 20 minutes |
| P+C+BL+ | P123 micelles with CUR | Blue light 20 minutes |
| C+BL+ | Free CUR | Blue light 20 minutes |
| M+C+UL+BL+ | PRP micelles with CUR | UV 1 hour + Blue light 20 minutes |

# Supplementary Figures and Tables

## Supplementary Figures


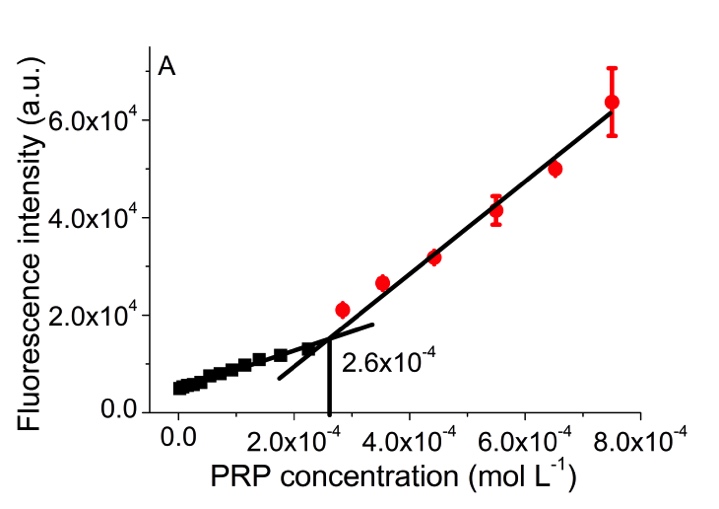

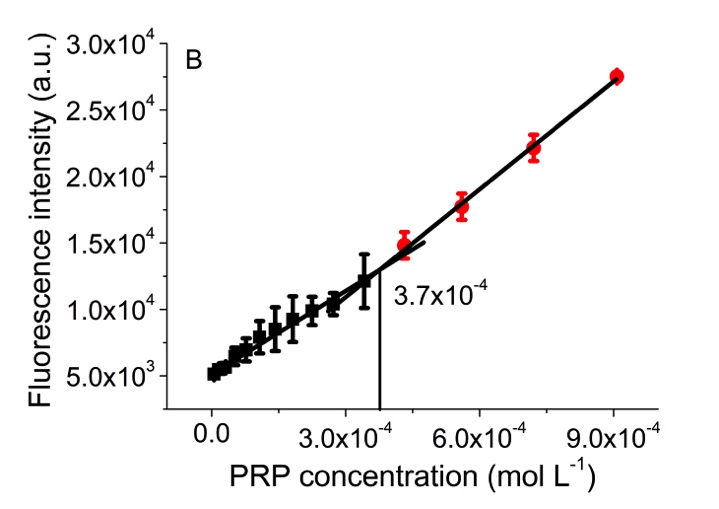


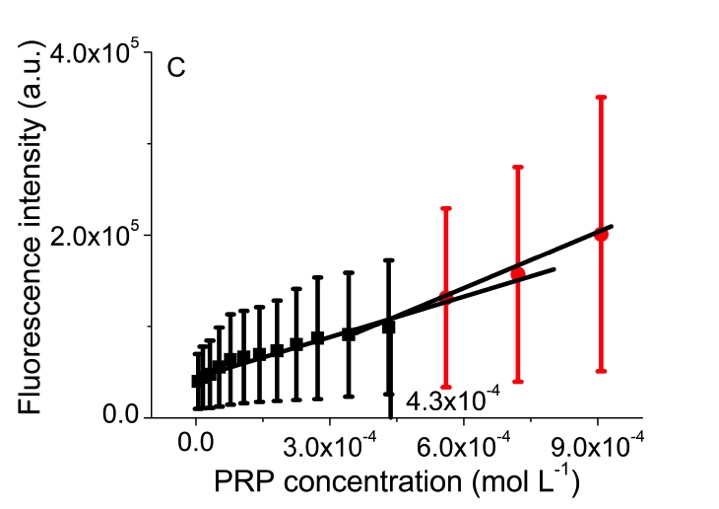


**Figure S1.** Critical Micellar Concentration of PRP in presence of pyrene at temperatures of: (A) 25 °C, (B) 30 °C, and (C) 35 °C.


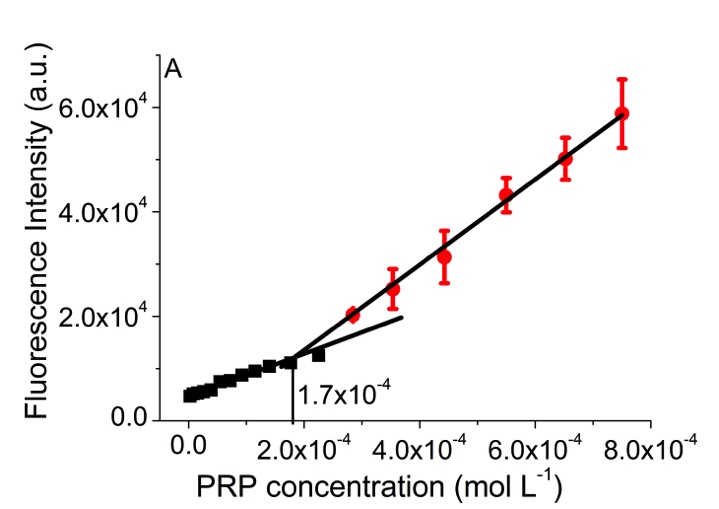

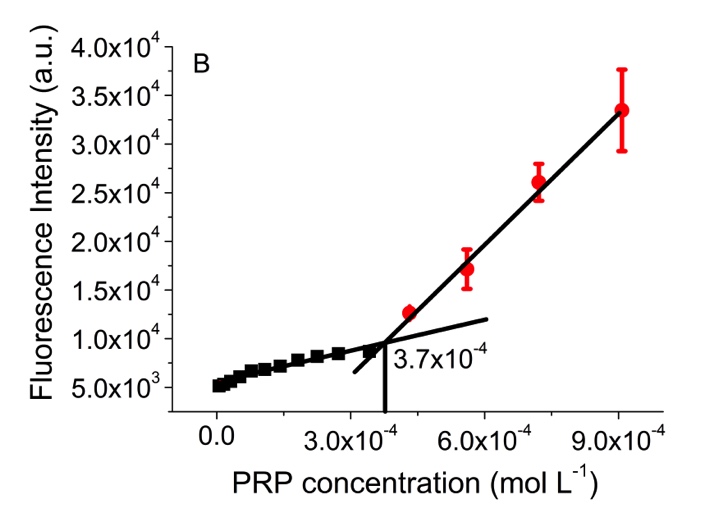


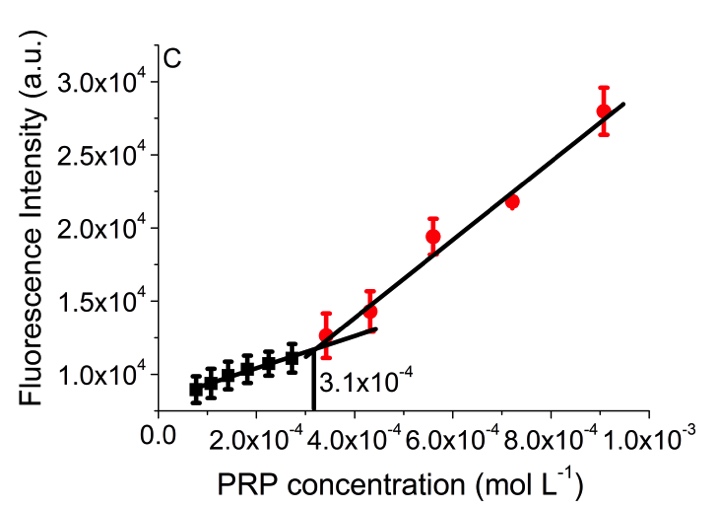


**Figure S2.** Critical Micellar Concentration of PRP in presence of CUR at temperatures of: (A) 25 °C, (B) 30 °C, and (C) 35 °C.


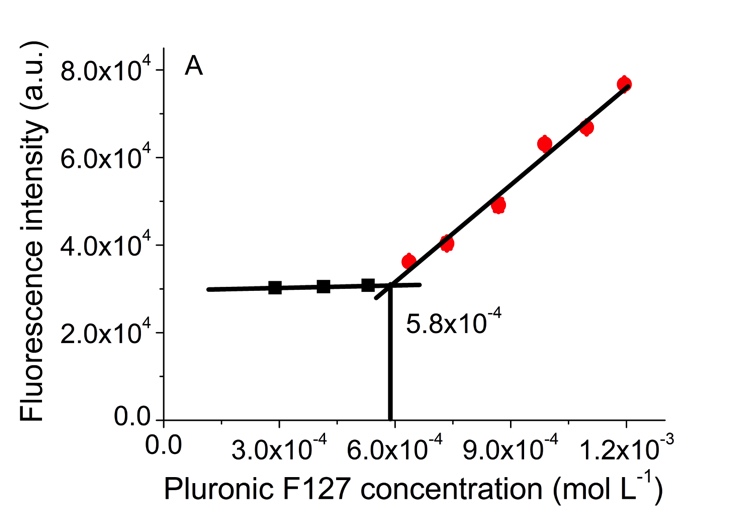

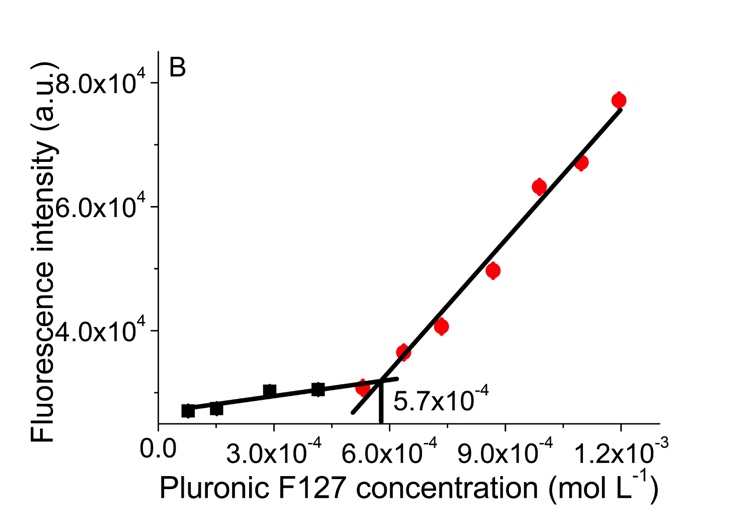

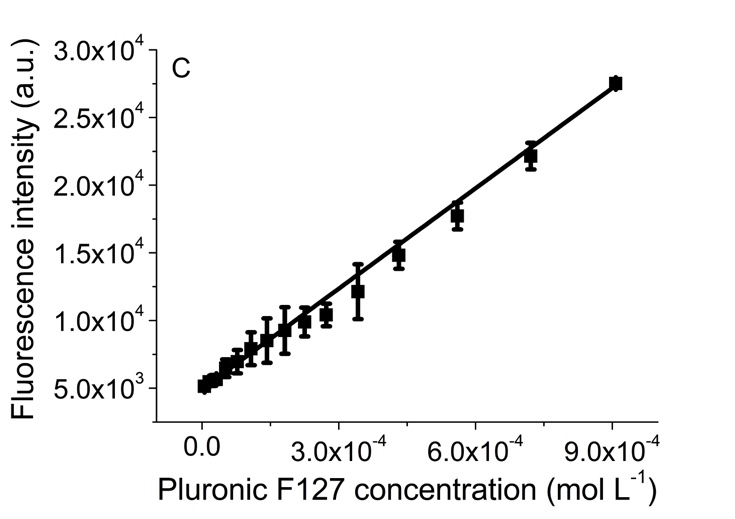

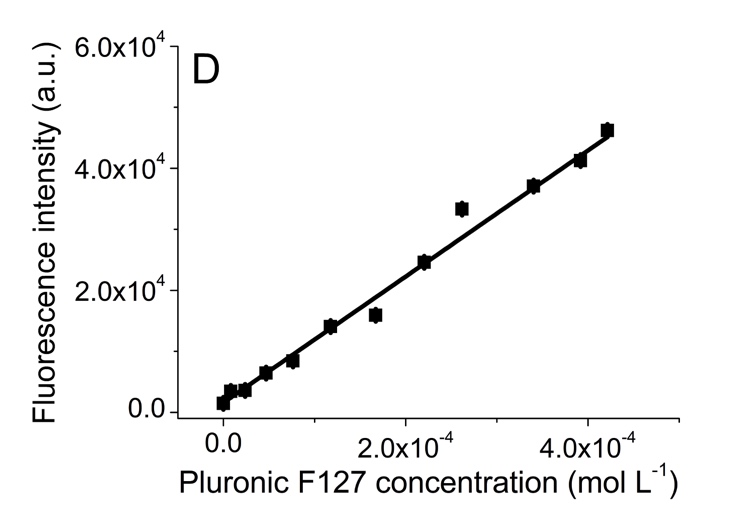


**Figure S3.** Critical Micellar Concentration of Pluronic F127 at: (A) 25 °C with CUR, (B) 25 °C without CUR, (C) 37 °C with CUR, (D) 37 °C without CUR.


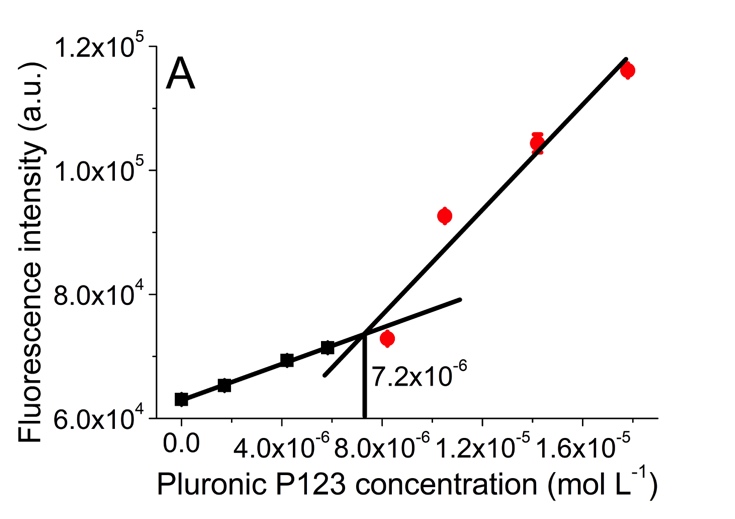

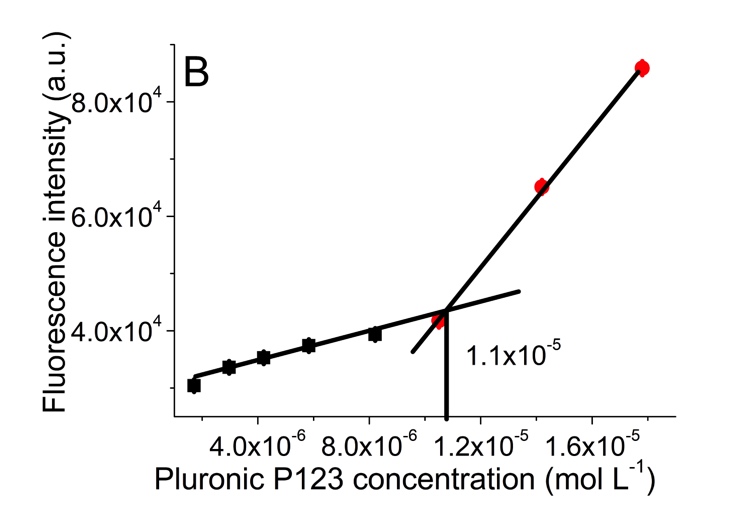


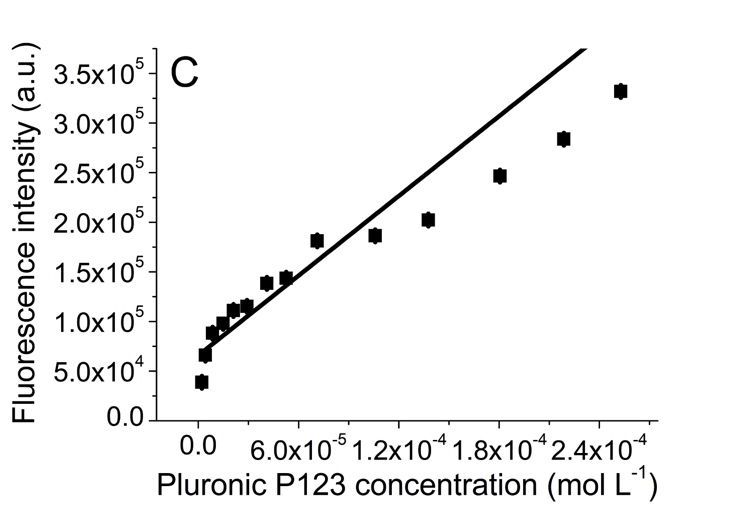

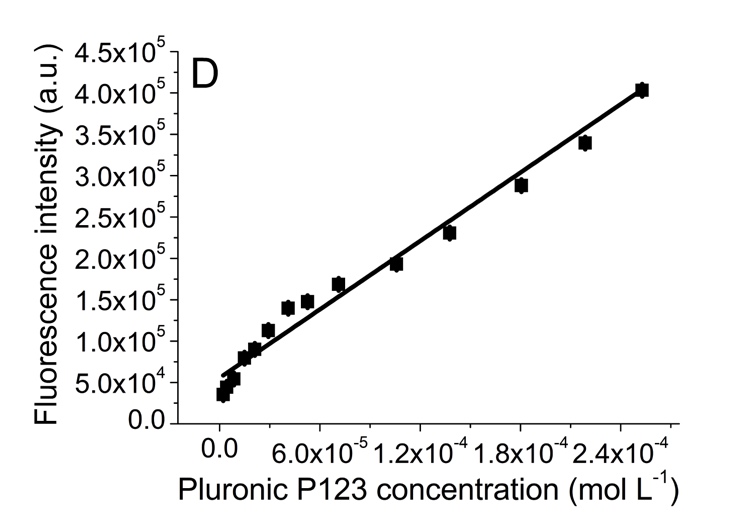


**Figure S4.** Critical Micellar Concentration of Pluronic P123 at: (A) 25 °C with CUR, (B) 25 °C without CUR, (C) 37 °C with CUR, (D) 37 °C without CUR.


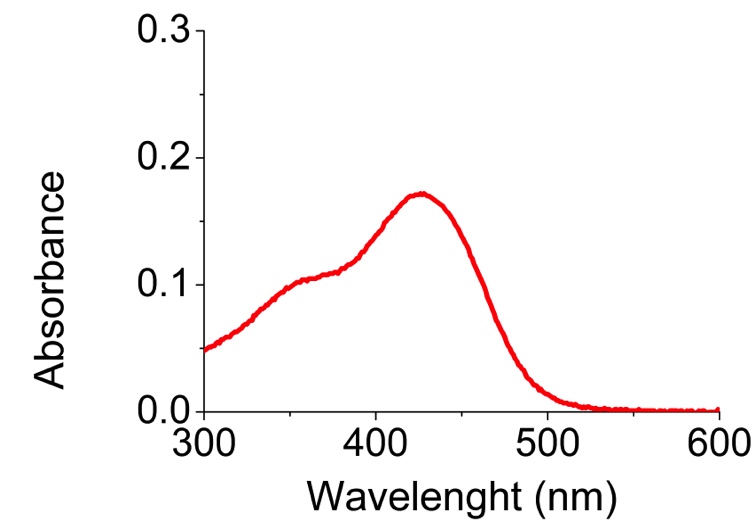


**Figure S5.** Absorption spectra of CUR in water.


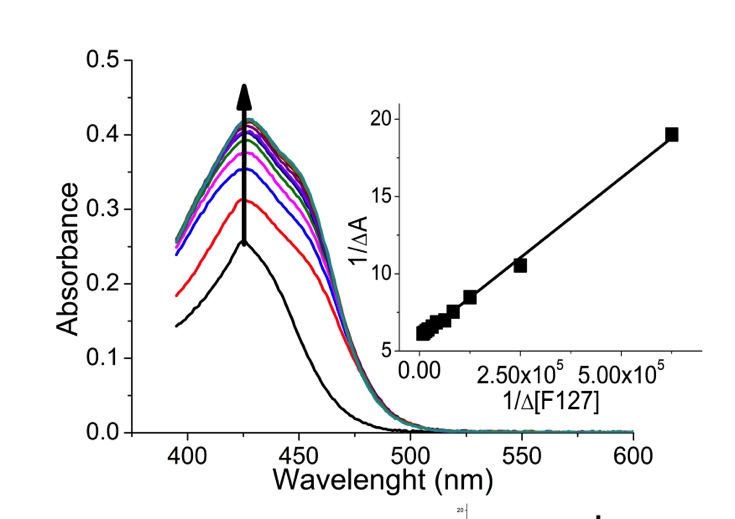

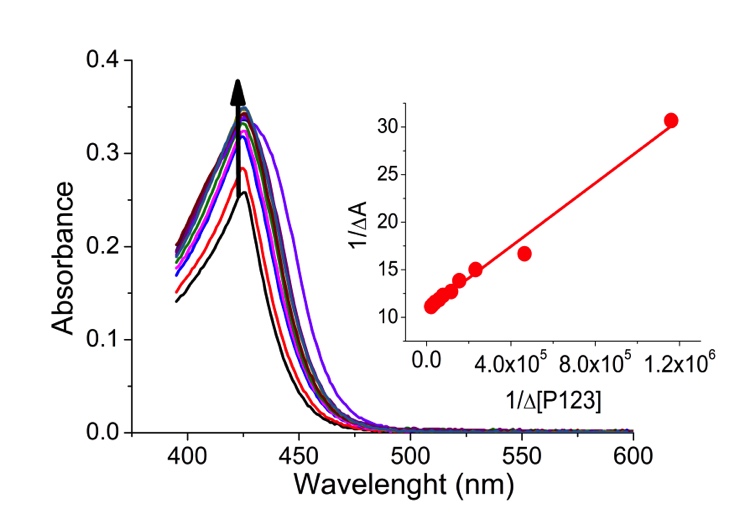


**Figure S6.** Absorption spectra and Ks linear fit from micelles synthesized with CUR: F127 micelles (black squares) and P123 micelles (red circles).


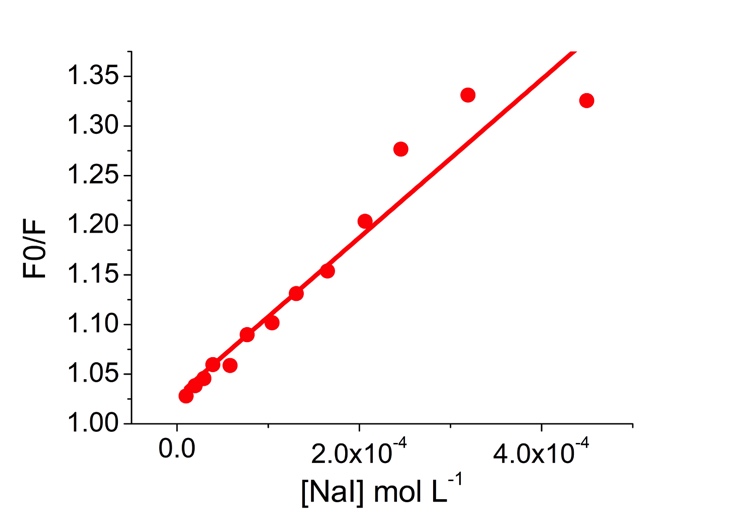

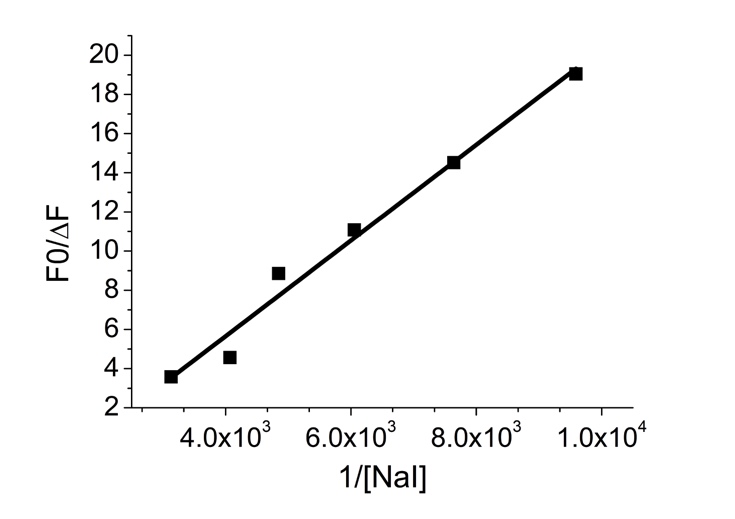


**Figure S7.** Fluorescence quenching of CUR in micelles of: P123 (red circles) and F127 (black squares).


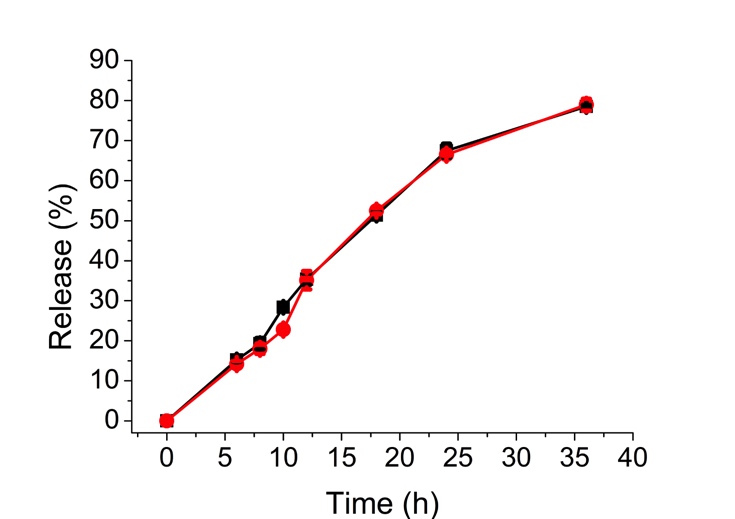


**Figure S8.** Release of CUR from F127 (black squares) and P123 (red circles) micelles.

**Figure S9.** Intracellular detection of Reactive Oxygen Species (ROS) of MRSA (A), *P. aeruginosa* (B), and *C. albicans* (C) incubated with 2',7’-dichlorofluorescein diacetate (DCFHDA) at 10 μM for 60 min. Microbial cells were submitted to aPDT (red bars) mediated by CUR in its free form or loaded into micelles (F127, P123, or PRP) or only incubated with the photosensitizer in the dark (blue bars) before measuring the fluorescence intensity of DCFHDA (details described in the section 2.5.1. of Materials and Methods). No significant difference (p>0.05) was observed among the groups. Error bars: standard deviation (n = 5).


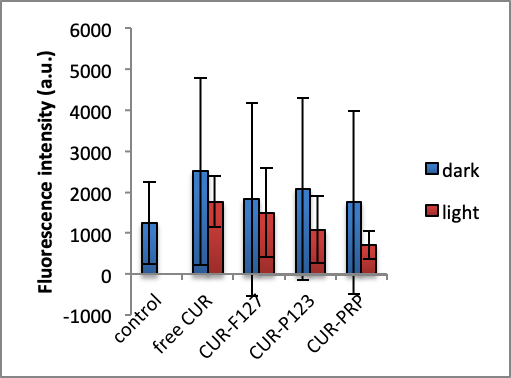

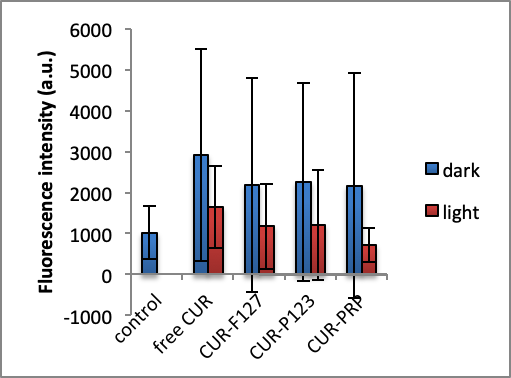

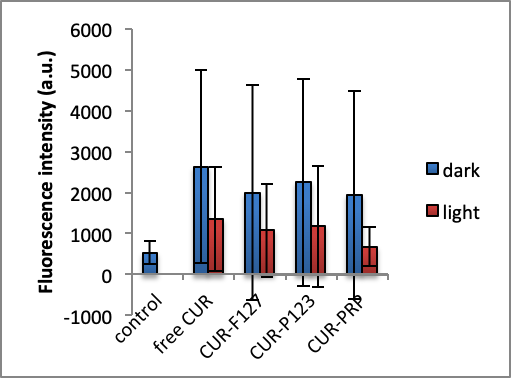


A

B

C

## Supplementary Tables

**Table S1.** Values of fluorescence intensity (arbitrary units) for intracellular detection of Reactive Oxygen Species (ROS) using 2',7’-dichlorofluorescein diacetate (DCFHDA) at 5 mM after aPDT* mediated by CUR in its free form and loaded into the micelles. Experiments were performed without and with centrifugation after incubating microbial samples with DCFHDA and before aPDT.

|  | Non-centrifuged | | | Centrifuged | | |
| --- | --- | --- | --- | --- | --- | --- |
|  | MRSA | *P. aeruginosa* | *C. albicans* | MRSA | *P. aeruginosa* | *C. albicans* |
| Without DCFHDA | 1278 | 2528 | 118 | 305 | 56 | 384 |
| Without PS | 2887 | 187592 | 510 | 7030 | 51792 | 2884 |
| CUR* | 1169 | 27525 | 266 | 1250 | 1118 | 1057 |
| CUR in F127* | 968 | 88063 | 83 | 539 | 389 | 401 |
| CUR in P123* | 429 | 80160 | 79 | 354 | 277 | 289 |
| CUR in PRP* | 1009 | 79377 | 135 | 610 | 536 | 524 |

*: parameters of light irradiation are described in the section 2.5.1. of Materials and Methods.

**Table S2.** Mean values of the hydrodynamic diameter (HD), PdI, and zeta potential of photo-responsive polymer (PRP), F127, and P123 micelles measured at 25ºC (parenthesis: standard deviation).

| Micelles | Without curcumin | | | With curcumin | | |
| --- | --- | --- | --- | --- | --- | --- |
|  | HD (nm) | PdI | Zeta potential (mV) | HD (nm) | PdI | Zeta potential (mV) |
| F127 | 19.3 (0.9) | 0.392 (0.245) | –0.61 (0.42) | 22.9 (1.2) | 0.333 (0.119) | –0.23 (0.29) |
| P123 | 16.1 (0.5) | 0.148 (0.075) | –0.22 (0.74) | 20.8 (1.6) | 0.249 (0.106) | –0.24 (0.27) |
| PRP | 31.9 (0.4) | 0.231 (0.481) | –0.15 (0.05) | 40.4 (0.8) | 0.271 (0.019) | –0.14 (0.01) |

**Table S3.** Entrapment efficiency (EE%), equilibrium constant (KS), and fluorescence quenching (K_SV_) of CUR. Measurements were acquired at 25 ºC (parenthesis: standard deviation).

| CUR form | EE% | K_S_ (×10^5^ L/mol) | R^2^ | K_SV_ (L/mol) | R^2^ |
| --- | --- | --- | --- | --- | --- |
| Free | - | - | - | 986 | 0.9697 |
| PRP micelles | 89 (1) | 1.00 | 0.9358 | 506 | 0.9763 |
| F127 micelles | 78 (2) | 2.83 | 0.9996 | 175 | 0.9877 |
| P123 micelles | 73 (1) | 6.82 | 0.9851 | 797 | 0.9375 |

-: not performed.

**Table S4.** CMC mean values of the photo-responsive polymer (PRP) were expressed in M with pyrene or CUR at four different temperatures (parenthesis: standard deviation).

| Temp. (ºC) | CMC (×10^-4^ mol/L) | |
| --- | --- | --- |
|  | PRP without CUR | PRP with CUR |
| 22 | 0.6 (0.1) | 0.6 (0.3) |
| 25 | 2.6 (0.3) | 1.7 (0.8) |
| 30 | 3.7 (0.5) | 3.7 (2.9) |
| 35 | 4.3 (3.7) | 3.1 (0.9) |

**Table S5.** Values of biofilms’ thickness (μm) and fluorescence intensity (arbitrary units, a.u.) emitted by each biofilm incubated with PBS (control) or CUR in its free form or loaded into the micelles (F127, P123, or PRP) evaluated on confocal microscopy.

|  | MRSA | *P. aeruginosa* | *C. albicans* |
| --- | --- | --- | --- |
| Control (untreated) | 702.080 a.u.  50 μm | 1,748.161 a.u.  80 μm | 741.290 a.u.  125 μm |
| Free CUR | 4,397.698 a.u.  70 μm | 1,458.407 a.u.  50 μm | 560.529 a.u.  100 μm |
| CUR in F127 micelles | 841.180 a.u.  60 μm | 1,821.827 a.u.  60 μm | 6,052.537 a.u.  130 μm |
| CUR in P123 micelles | 3,160.101 a.u.  70 μm | 4,992.395 a.u.  70 μm | 5,692.543 a.u.  170 μm |
| CUR in PRP micelles | 921.830 a.u.  60 μm | 1,098.460 a.u.  50 μm | 1,629.563 a.u.  280 μm |
